# Illuminating the inner workings of a natural protein switch: Blue-light sensing in LOV-activated diguanylate cyclases

**DOI:** 10.1126/sciadv.adh4721

**Published:** 2023-08-02

**Authors:** Uršula Vide, Dženita Kasapović, Maximilian Fuchs, Martin P. Heimböck, Massimo G. Totaro, Elfriede Zenzmaier, Andreas Winkler

**Affiliations:** ^1^ Institute of Biochemistry, Graz University of Technology, Petersgasse 12/II, 8010 Graz, Austria.; ^2^ BioTechMed-Graz, Mozartgasse 12/II, 8010 Graz, Austria.

## Abstract

Regulatory proteins play a crucial role in adaptation to environmental cues. Especially for lifestyle transitions, such as cell proliferation or apoptosis, switch-like characteristics are desirable. While nature frequently uses regulatory circuits to amplify or dampen signals, stand-alone protein switches are interesting for applications like biosensors, diagnostic tools, or optogenetics. However, such stand-alone systems frequently feature limited dynamic and operational ranges and suffer from slow response times. Here, we characterize a LOV-activated diguanylate cyclase (LadC) that offers precise temporal and spatial control of enzymatic activity with an exceptionally high dynamic range over four orders of magnitude. To establish this pronounced activation, the enzyme exhibits a two-stage activation process in which its activity is inhibited in the dark by caging its effector domains and stimulated upon illumination by the formation of an extended coiled-coil. These switch-like characteristics of the LadC system can be used to develop new optogenetic tools with tight regulation.

## Teaser

A two-stage regulation mechanism enables high dynamic ranges of enzyme activation in a light-controlled protein switch.

## INTRODUCTION

Adapting to environmental cues in unicellular organisms and integrating cellular signals from distant parts of higher organisms requires the action of specialized regulatory proteins. Depending on the evolutionary imprinting of protein properties in diverse signaling cascades, functional outputs can feature either linear dose-response curves or switch-like characteristics (*1*). Especially for lifestyle-defining transitions, proliferation, or cell death, all-or-nothing responses are desirable. In other words, only if a certain threshold is exceeded will the full response be observed for the regulated functionality.

Since such on-off switch characteristics are rather challenging to realize for isolated protein-based systems, the regulation of specific activities is frequently coupled to accessory proteins that either keep the central protein’s activity off or further stimulate it once activated. Well-known examples of such a control mechanism are small guanosine triphosphatases (GTPases) that regulate differentiation, transport, or cell growth with switch-like characteristics enabled by the concerted action of GTPase-activating proteins and nucleotide-exchange factors (*2*). Additional options to achieve ultrasensitivity in signal processing, in other words switch-like properties, are cooperativity, positive feedback loops, multisite phosphorylation, or signaling cascades (*3*–*5*).

Irrespective of how the switch-like properties are achieved on a molecular level, specific characteristics of these systems render them interesting for applications such as biosensors, diagnostic tools, or optogenetics. For example, a fast response time (transition time between on and off conformations) is beneficial for protein conformational switches in the field of biosensors (*6*). Similarly, a high dynamic range (defined as the range between minimal and maximal output activities) is desirable for orthogonal applications in which signaling elements cannot build upon the amplification of endogenous regulatory circuits (*7*, *8*). Last but not least, the operational range (the input concentration range that is able to affect output activity) needs to be tailored to the requirements of specific applications. While in nature evolution has typically optimized these properties for the specific requirements of a signaling circuit, non-natural applications of switches frequently suffer from limitations in control over spatial distributions of chemicals within organisms or limitations in temporal control of turning a signal on and off again.

One powerful alternative to activation by chemical cues is the possibility of using light as the actuator of a signaling cascade. In the same way that nature uses photoreceptors to control a plethora of biological activities (*9*), scientists are exploiting the beneficial temporal and spatial control mechanisms of light in the field of optogenetics (*10*). To this end, both natural and artificial systems are used in diverse fields of molecular biology (*11*). Since light actuation can be controlled very precisely in both time and space, the response time and the operational range are usually not limiting in optogenetics. However, the dynamic range of many photoreceptors and optogenetic tools is limited and typically not straightforward to optimize by engineering efforts.

Here, we present a blue-light sensor coupled to a GGDEF effector domain (*12*) that features exceptionally high fold changes of activation upon illumination. The name of the latter domain refers to a highly conserved sequence motif in the active site of GGDEF domains that enables GTP coordination and eventually diguanylate cyclase activity (*12*). The blue-light sensor used in this system is a light-oxygen-voltage (LOV) domain, featuring a canonical flavin adduct upon blue light illumination (*13*). To address whether LOV-activated diguanylate cyclases (LadCs) use a similar helical register-switching mechanism involving two different coiled-coil architectures of the sensor-effector linker as proposed for phytochrome- and receiver-regulated diguanylate cyclases (*14*, *15*), we used an integrative structural biology approach. Thereby, we characterized molecular details of how the more than 10,000-fold stimulation of diguanylate cyclase activity is realized by the coupled LOV-sensory domain (*13*) and identified a more complex regulatory mechanism. The combination of a dark-state crystal structure with the analysis of solution scattering data and conformational dynamics in the illuminated state revealed a two-stage activation mechanism. On the one hand, full inactivation of enzymatic activity in the dark is achieved by caging of the GGDEF domains on opposite sides of the LOV dimer architecture. On the other hand, an extended dimer conformation formed by the coiled-coil sensor-effector linker upon illumination stimulates productive encounter of the GGDEF domains for the efficient formation of the bacterial second-messenger cyclic–dimeric–guanosine 3′,5′-monophosphate (c-di-GMP).

These switch-like characteristics, observed for a single soluble protein, and the detailed mechanistic descriptions on a molecular level open up new design strategies for powerful optogenetic tools based on the LadC system and its dual-stage activation process. Since no accessory proteins are needed and blue-light sensing requires only the ubiquitous flavin cofactor, the system will be readily accessible in a variety of biological backgrounds (*16*).

## RESULTS

**Fig. 1.**
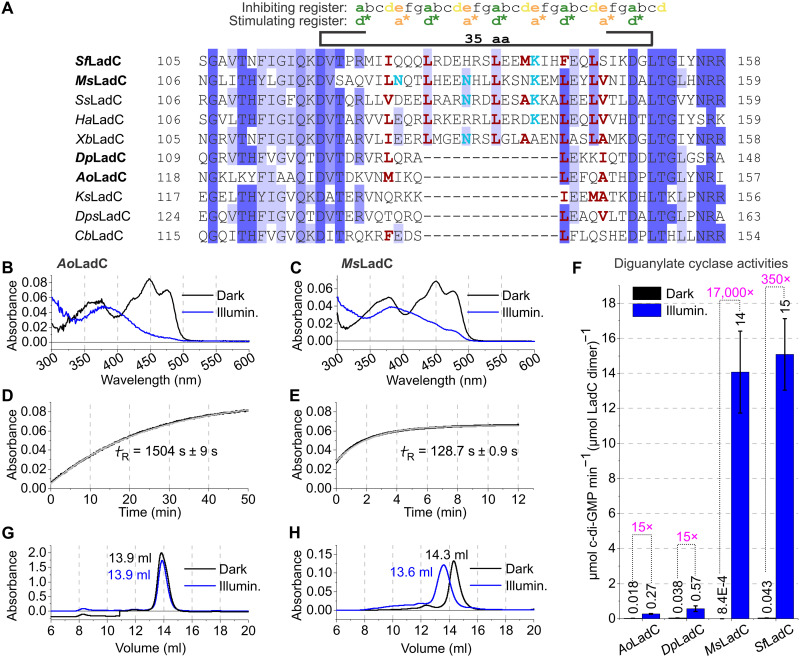
Alignment of homologous LOV-GGDEF protein sequences and biochemical characterization of four LadC homologs. (**A**) Close-up of the sensor-effector linker region in the alignment of LadC homologs featuring all characteristic motifs for LOV and GGDEF domains. Sequences are grouped based on the two linker length-derived subfamilies. LadC homologs characterized in this work are highlighted with their names in bold. Two possible heptad-repeat arrangements of the linker are shown on top, and important residues are colored in green, yellow, and orange. The color scheme of highlighted residues in the alignment corresponds to the degree of conservation based on the full alignment shown in fig. S1. Hydrophobic residues at positions *a*, *d*, and a* are represented in bold ruby-red if they are conserved in over 50% of the linker cluster. Asn and Lys residues in position a* are represented in bold turquoise. (**B** and **C**) UV-visible (UV-Vis) absorption spectra of *Ao*LadC and *Ms*LadC, each representing one of the two linker subfamilies. (**D** and **E**) Dark-state recovery kinetics of *Ao*LadC and *Ms*LadC at 20°C with the respective single exponential fits in gray. Mean lifetimes and the SE of the estimate from the fit are indicated. (**F**) Characterization of enzymatic activities of four LadC homologs. Dark-state activities are shown in black and light-state activities in blue. Sample SDs of triplicates measurements contributed to the error estimation of the linear fit used to calculate the initial rate of product formation. The SE of the linear regression estimate is shown as an error bar. (**G** and **H**) Gel filtration chromatograms of *Ao*LadC and *Ms*LadC, respectively. Shown are 280-nm absorbance traces. aa, amino acid.

We extracted protein sequences featuring a sensory LOV domain followed by a GGDEF effector domain from Basic Local Alignment Search Tool (BLAST) and Position-Specific Iterated BLAST (PSI-BLAST) searches and identified two LadC clusters based on the length of the sensor-effector linker region (Fig. 1A). The linker length between the two clusters differs by 14 amino acid residues. In the cluster with the longer linker, the linker between the LOV and GGDEF domains is 35 amino acid residues long, as defined by the peptide sequence starting with the conserved Asp in the D(I/V)(T/S) motif of the LOV domain and ending with the conserved Leu in the DxLT motif of the GGDEF domain. Furthermore, the linker region in both clusters displays characteristics of heptad-repeat patterns, suggesting a coiled-coil architecture (*17*). To address the potential helical register-switching mechanism observed for diguanylate cyclases regulated by different sensory inputs (*14*, *15*) and to understand how characteristic linker-length differences in the two LadC clusters affect this regulation, we expressed and purified selected members of the two LadC subfamilies. On the basis of their origins, the proteins are referred to as *Ms*LadC (*Methylotenera sp.*) and *Sf*LadC (*Serratia fonticola* AU-AP2C), *Ao*LadC (*Aquella oligotrophica*) and *Dp*LadC (*Deinococcus peraridilitoris* DSM 19664) (Fig. 1A). *Ms*LadC and *Sf*LadC contain long linkers (35 amino acids), whereas *Ao*LadC and *Dp*LadC belong to the short linker subfamily (21 amino acids). Full sequence alignments and additional protein sequences with different linker lengths but lacking typically conserved residues in the LOV domain (see the “Protein expression and purification” section) are provided in fig. S1.

### Biochemical characterization

All LadC homologs were purified via Nickel-Nitrilotriacetic acid (Ni-NTA) chromatography, followed by His-GB1-tag removal and a subsequent inverse Ni-NTA step. A final polishing step via gel filtration was included to isolate the proteins of interest. All characterized LOV-GGDEF homologs show a typical dark-state absorption spectrum of the LOV-bound oxidized flavin, as well as a characteristic spectrum for its C4a-cysteinyl adduct following activation with blue light (Fig. 1, B and C, and fig. S2, A and B). The thermal recovery kinetics of the flavin adduct at 20°C range from a few minutes, for *Ao*LadC and *Ms*LadC (Fig. 1, D and E), to several hours, for *Sf*LadC and *Dp*LadC (fig. S2, C and D), with no apparent correlation to the linker length. *Sf*LadC’s thermal recovery displays a two-phasic trend. Since *Sf*LadC was the only LadC homolog in this study that showed multiple oligomeric species in solution, especially upon illumination (see below and fig. S2E), one potential explanation for the more complex recovery kinetics could be oligomeric state-dependent differences in stability of the light-state conformation. As far as diguanylate cyclase activity of all characterized LadC homologs is concerned, we observed an apparent up-regulation upon illumination with blue light (Fig. 1F), with pronounced differences between the representatives of the two linker length families. While the short linker members featured moderate fold changes on the order of 15-fold upon activation, the longer linker systems exhibited substantially higher fold changes reaching beyond 10,000-fold increases in specific activity upon illumination (Fig. 1F). As far as dynamic ranges are concerned, the difference can be accounted for by a very tight dark state inhibition together with pronounced higher catalytic efficiency in the light state of the longer linker representatives that provide the switch-like characteristics observed for *Sf*LadC and *Ms*LadC. The importance of tight dark state inhibition becomes more evident under reaction conditions that are better suited for short linker representatives, where they feature high activities in the dark as well as in the light state (fig. S3, A and B). Long linker representatives, however, remain tightly inhibited in their non-activated state throughout all tested conditions (fig. S3, C and D). To address functional properties of the inhibited conformation and the high dynamic ranges associated with it, we focused on experimental settings most suitable for *Ms*LadC. Under the experimental conditions of the activity assays, the homologs *Ao*LadC, *Dp*LadC, and *Ms*LadC did not only produce c-di-GMP but also the reaction intermediate pppGpG, which is formed by a single phosphodiester linkage between two GTP substrate molecules and typically only accumulates if the GGDEF protomers are not optimally positioned for formation of the second bond leading to c-di-GMP (*18*, *19*). Tests with different reaction conditions (fig. S3) revealed that the ratio between c-di-GMP and pppGpG depends on several parameters, such as the substrate concentration, temperature, and protein concentration (fig. S3, E and F). In addition, *Ms*LadC (fig. S3G), *Sf*LadC and *Dp*LadC exhibited allosteric product inhibition at higher substrate concentrations, consistent with the observation in the majority of GGDEF enzymes and the presence of the RxxD inhibitory site (*12*). However, at a GTP concentration of 50 μM and addressing initial velocities of product formation with a maximum turnover of 10% no influence of product inhibition is observed, enabling a comparative analysis of all tested constructs (Fig. 1F). *Ao*LadC completely lacks the RxxD motif (fig. S1), which results in a linear increase of product formation in the progress curve (fig. S3H) also at higher substrate concentrations.

### In-solution light-induced structural changes

Analytical size-exclusion chromatography (SEC) runs of all characterized LadC homologs under non-actinic light conditions revealed species corresponding to dimeric LOV-GGDEF assemblies. However, upon illumination, long linker length LadCs showed shifts in their elution profile that could be induced by either pronounced structural rearrangements or changes in oligomerization. By determining the absolute molar mass with a SEC-coupled multiangle light scattering (MALS) detector (table S1) and comparing the retention volumes (Fig. 1, G and H, and fig. S2, E and F), we showed that all characterized LadC homologs assemble as homodimers in solution in the dark as well as in the illuminated state. However, the peaks of both *Ms*LadC and *Sf*LadC shifted toward smaller retention volumes upon illumination (Fig. 1H and fig. S2E), indicating that the illuminated state might feature characteristic structural rearrangements compared to the dark-state structure. An earlier elution of an illuminated dimer is indicative of an elongated ellipsoid shape relative to a more compact dark-state conformation. For *Sf*LadC, an additional and reversible shift toward higher oligomeric species occurred under blue light illumination (fig. S2E). While the dimeric assembly of both homologs with the long linker is similarly affected by illumination, the short linker LadC homologs showed no difference in retention behavior upon illumination (Fig. 1G and fig. S2F).

### Crystal structure of a full-length dark-state LadC

**Fig. 2.**
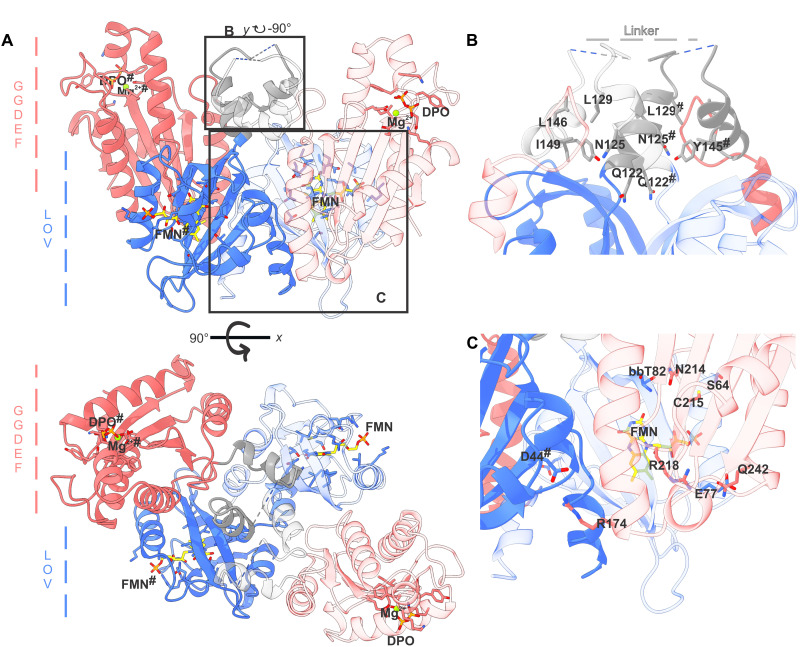
Crystal structure of dimeric *Ms*LadC in its dark-adapted state. (**A**) Cartoon representation with LOV domains in blue colors, linker helices in gray, and GGDEF domains in red, and one protomer distinguished in transparency. The FMN cofactor (yellow), pyrophosphate (orange), and a Mg^2+^ ion (green) and their binding sites are shown as stick models. (**B** and **C**) Close-up view of the inhibitory interactions in the linker and the inhibitory interface interactions between LOV and GGDEF domains, respectively. Potential interacting residues are shown as stick models. Superscript hashtags denote elements of the respective other protomer in the dimeric assembly. More details and electron densities are provided in fig. S4. bb, backbone.

The question of whether the linker length and/or its composition is essential for enabling tight inhibition of the GGDEF domains under non-actinic conditions in long linker LadC homologs was addressed by elucidating the crystal structure of full-length *Ms*LadC in its dark-adapted state (Fig. 2A, fig. S4A, and table S2). The structure was solved by a combination of molecular replacement and single-wavelength anomalous diffraction (MR-SAD) phasing. However, structure solution was not straightforward due to atypical intensity distributions of the observed reflections, which was reproducibly present for datasets deriving from crystals originating from different protein batches, different data acquisition protocols, or varying crystallization conditions (fig. S4B). Details of the structure solution and refinement protocols are described in Materials and Methods. One *Ms*LadC molecule is present in the asymmetric unit with bound riboflavin 5′-phosphate (FMN) (fig. S4C), pyrophosphate, and a Mg^2+^ ion (fig. S4D). The pyrophosphate was apparently copurified from the bacterial expression cultures, while FMN was added in excess during the purification process and Mg^2+^ was included as part of the purification buffers. We built the entire LOV and GGDEF domains into the electron density map, missing only 4 disordered residues in the middle of the linker region (fig. S4E), 10 residues at the flexible N terminus, and 2 at the C terminus. Visualization of symmetry mates revealed a parallel dimeric assembly of *Ms*LadC as the biological unit consistent with its in-solution behavior. Proteins, Interfaces, Structures and Assemblies (PISA) analysis supports this assembly with a large buried surface area between the two protomers (2785 Å^2^, ignoring ligand contacts) and high free energy of assembly dissociation (14.2 kcal/mol) (*20*). Overall, the structure reveals a characteristic LOV-dimer interdomain interface (*21*–*23*), comprising the A′α helices and the core β sheet of the LOV domain. Residues in the chromophore binding pocket that interact with FMN comply with the residues previously identified at its *si*- and *re*-face (*24*–*26*). The structure reveals a tight interaction of the LOV domains with their C-terminally linked GGDEF domains. Rather than forming an extended coiled-coil linker, the linker region is disordered between Glu^131^ and Asn^139^, and the GGDEF domain folds back onto the LOV domain (fig. S4E). Hydrophobic interactions between Leu^129^, Leu^146^, and Ile^149^ or hydrogen bonding between Asn^126^ and Tyr^145^ potentially stabilize such a bent and inhibited architecture within the linker region (Fig. 2B). In addition, the inhibitory interface is formed between the surfaces of the LOV and GGDEF domains. For example, Asp^44#^ (hashtag denotes the adjacent protomer) from the LOV’s Cα-Dα loop forms a salt bridge with Arg^174^ from the GGDEF’s α^0^ helix (Fig. 2C), and Glu^77^ from the LOV’s Fα-Gβ loop interacts with Gln^242^ from the GGDEF’s α^3^ helix (Fig. 2C). Also, the GGDEF’s α^2^-β^2^ loop, containing the RxxD inhibitory site (*12*), is packed against the LOV core (Fig. 2C, Arg^218^). In addition, Asn^214^ and Cys^215^ from the GGDEF’s α^2^-β^2^ loop interact with the backbone of the LOV’s Gβ-strand and Ser^64^ of the Fα helix, respectively (Fig. 2C). The buried surface area of the inhibitory interface in the dimeric assembly amounts to 3073 Å^2^, as calculated using PISA (*20*). This interaction interface, together with interactions within the linker region, explains the inactive, “switched off,” dark state. Facilitated through numerous specific interactions, the inhibited state prevents the formation of the anticipated continuous coiled-coil linker helices, which apparently provide enough structural flexibility and the appropriate length for the observed inhibitory conformation. On the basis of the crystal structure and the conserved residues in the linker region (Fig. 2A and fig. S1), LadCs with shorter linkers cannot fold into the inhibitory conformation due to steric incompatibility with the tight arrangement of parts of the linker and the wide turn at the beginning of the GGDEF domain.

### AlphaFold2 predictions

**Fig. 3.**
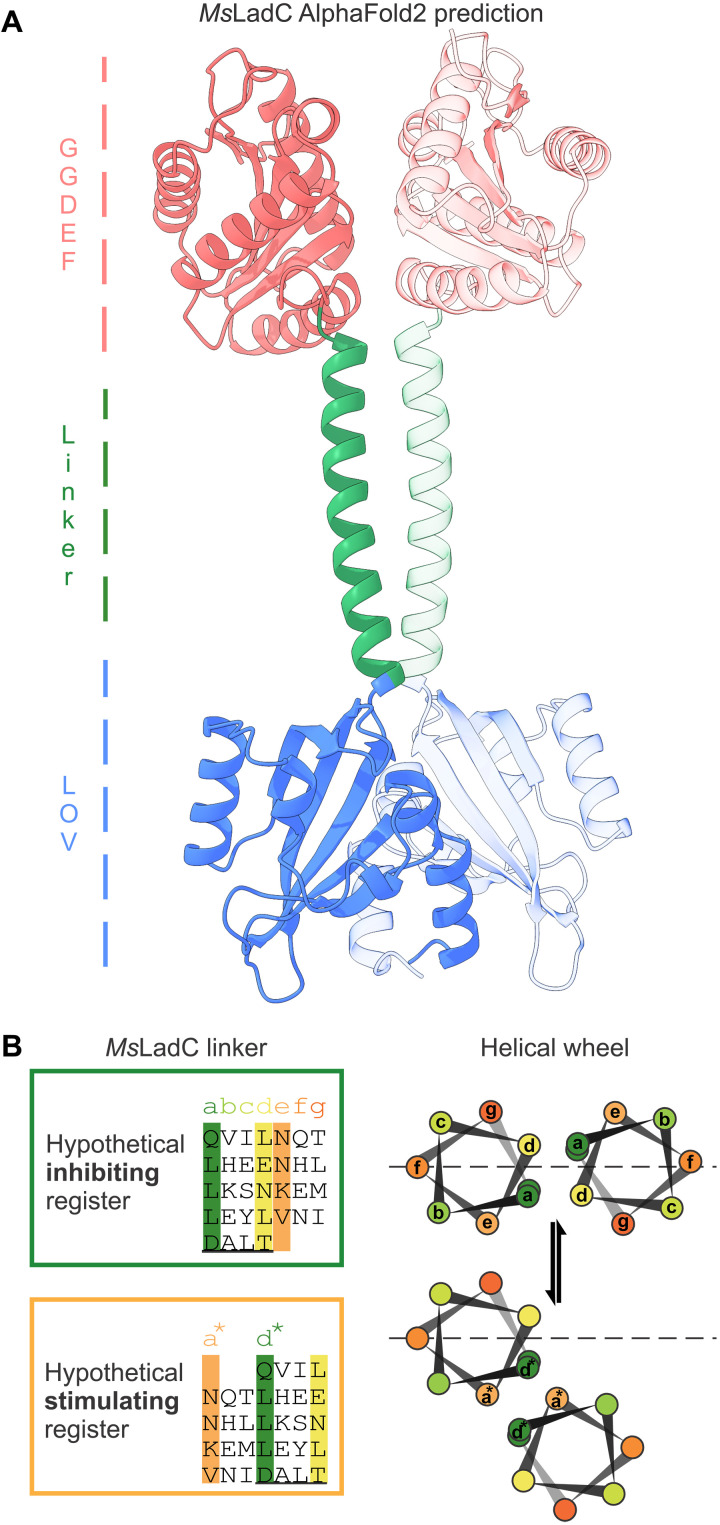
*Ms*LadC structure prediction by AF2 and hypothetical coiled-coil registers of the sensor-effector linker. (**A**) Elongated model with linker helices in a coiled-coil architecture as predicted by AF2. The corresponding Predicted Aligned Error (PAE) plot is shown in fig. S5D. (**B**) *Ms*LadC linker residues in the AF2 model align with the hypothetical inhibiting register. The hypothetical stimulating register is expected in an enzymatically active light-state conformation (*14*). Underlined amino acid residues correspond to the conserved N-terminal motif of the GGDEF domain (DxLT, underlined). Corresponding helical wheel translation was modified from (*35*).

The continuous coiled-coil architecture of the linker region is not only indicated by the conservation of linker length and the heptad repeat pattern in many naturally occurring LadC sequences but also suggested by AlphaFold2 (AF2) models (Fig. 3A and fig. S5). As shown in Fig. 3A, the AF2 model of *Ms*LadC, created by enforcing a dimeric assembly, reveals the characteristic LOV dimer similar to that observed in the crystal structure. In contrast to the crystal structure, however, an ideal coiled-coil architecture of the sensor-effector linker is predicted in the AF2 model, which results in the GGDEF domains being positioned close to each other as would be required for catalysis to proceed (*12*). It should be pointed out that no flavin cofactor is present during model generation and that no light condition can be specified. Therefore, the models generated reflect the conformational space accessible to the proteins solely based on their amino acid sequence. In analogy to the helical registers proposed for the Phytochrome-activated diguanylate cyclase (PadC) system (*14*), the AF2 model of *Ms*LadC shows the coiled-coil linker in an inhibiting-register conformation (Fig. 3B, top) as defined by the positioning of the DxLT motif relative to the heptad repeat units. The *a* and *d* positions of the heptads are mostly occupied by hydrophobic residues, but some destabilizing residues are also observed (Glu^132^ and Asn^139^ at the *d* position), flanking the disordered linker region of the crystal structure. Along this line, such a register is not observed in the dark-state crystal structure, as the inhibitory interface dominates the overall assembly. On the other hand, the hypothetical stimulating register also features nonhydrophobic residues at the a* position: Asn^126^, Asn^133^, and Lys^140^ (Fig. 3B, bottom). A functional role for the Asn residues at position a* has been observed in coiled-coils in general (*17*), as well as in stimulating registers of coiled-coils regulating diguanylate cyclases (*14*, *15*), and also Lys residues at this position can stabilize the specificity of the parallel dimeric assembly (*27*). AF2 predictions of the remaining LadC homologs also produced models exclusively featuring the coiled-coil linker architectures (fig. S5). As shown in fig. S5C, the *Dp*LadC prediction actually features the respective a* and d* residues at the coiled-coil interface defining the stimulating-register conformation. The observed translational rearrangement at the coiled-coil interface of the LadC AF2 models (Fig. 3 and fig. S5) is in line with observations for *Is*PadC and DgcR (*14*, *15*).

### Conformational dynamics (HDX-MS)

**Fig. 4.**
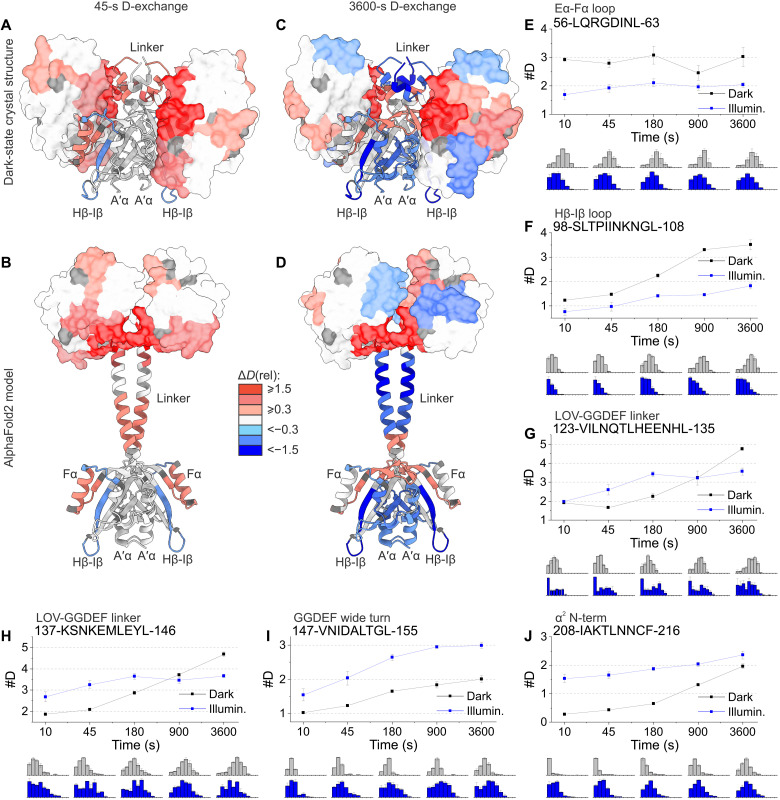
HDX-MS characterization of *Ms*LadC. (**A** to **D**) The *Ms*LadC dark-state crystal structure and the *Ms*LadC AF2 model are colored according to differences in deuterium uptake (Δ*D*_rel_) after 45 s or 1 hour of deuteration. LOV domains and linker helices are depicted as cartoon, and GGDEF domains are depicted as surface. (**E** to **J**) Deuterium uptake curves of selected *Ms*LadC peptides, with #D plotted against the deuteration time for dark- (black) and light-state (blue). Plotted are mean values of each peptide’s centroid value of three independent measurements, and error bars correspond to the sample SD. The plots underneath the deuterium uptake curves show the corresponding abundance distributions of deuterated species ranging from undeuterated to fully deuterated amide positions.

To address the conformational dynamics of *Ms*LadC and structural rearrangements upon illumination, we used the technique of hydrogen-deuterium exchange coupled to mass spectrometry (HDX-MS) that has proven powerful for the characterization of functionally important elements in photoreceptors (*14*, *28*, *29*). Figure 4 shows difference plots for *Ms*LadC comparing dark-adapted and blue-light–illuminated samples (see fig. S6 for full peptide map and the “Supplementary HDX Data” section in the Supplementary Materials). In Fig. 4 (A and C), we show differences in deuterium uptake plotted onto the dark-state crystal structure, which helps to visualize the increase in conformational dynamics for peptides lining the inhibitory LOV-GGDEF interface. Figure 4 (B and D) shows the same data plotted onto the AF2 model of *Ms*LadC, illustrating that the increased conformational dynamics in the GGDEF domain are likely a consequence of such an elongated arrangement. As indicated by the deuterium incorporation levels upon illumination (Fig. 4, E to J, blue plots), a decrease in conformational dynamics can be observed in regions of the LOV domain (Fig. 4, A to F), whereas an increase can be seen in several functionally important regions of the GGDEF domain (Fig. 4, A to D, I, and J). In the LOV domain, primarily the dynamics of the loop regions are affected, especially the Eα-Fα and Hβ-Iβ loops display reduced conformational dynamics upon illumination (Fig. 4, A to F). On the other hand, the Fα helix and the loop to the Gβ-strand show increased dynamics (Fig. 4, A to D). While differences in deuterium uptake for short labeling times typically reflect structural rearrangements, longer incubation time points reveal interesting insights into altered conformational dynamics of elements potentially involved in allosteric signal integration. The A′α helix of the LOV domain showed no significant changes in deuterium uptake upon illumination for early time points but reduced dynamics compared to the dark state became apparent at longer incubation (Fig. 4, C and D). An interesting observation was made for the highly conserved wide turn, with its DxLT motif at the GGDEF domain’s N terminus, and the subsequent α^0^ helix. They are both a central part of the inhibitory interface, as revealed by the crystal structure, and display pronounced changes in conformational dynamics at all incubation time points (Fig. 4, A to D and I). A substantial change in conformational dynamics upon illumination also occurred at the end of the α^2^ helix, which is packed against the LOV core in the inhibited dark state (Fig. 4J). The linker region generally showed slightly higher deuterium uptake levels for short incubation time points; however, during longer incubations, the incorporated deuterium levels were lower than those for the respective dark measurements (Fig. 4, A to D, G, and H). This points to more complex structural rearrangements in this region, which is also reflected in the bimodal distributions of the deuterium uptake upon illumination for the GGDEF wide turn and the linker region (Fig. 4, G to I, blue bottom plots); such observations are characteristic for a specific exchange regime (EX1) in Hydrogen/Deuterium exchange (*30*) that is typically observed for regions featuring distinct structural properties. This results in rapid deuterium uptake in one state and slower to moderate deuterium incorporation in the other state, due to the slower exchange kinetics between the two conformations.

### In-solution structure determination

**Fig. 5.**
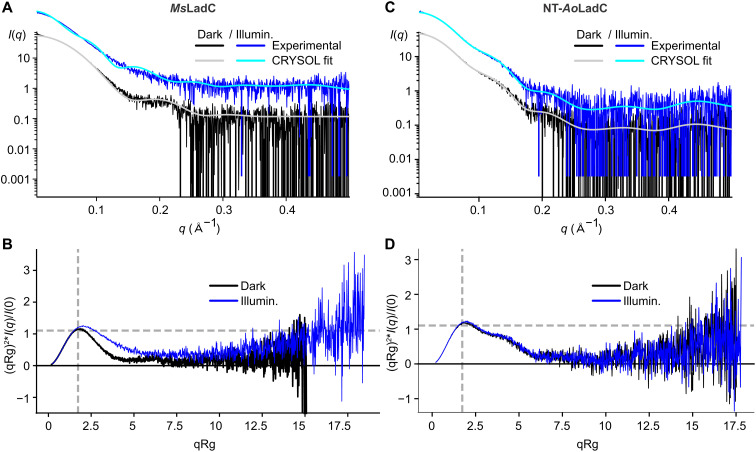
In-solution scattering of *Ms*LadC and NT-*Ao*LadC. Scattering curves of (**A**) *Ms*LadC and (**C**) NT-*Ao*LadC in dark- (black) and light-state (blue). Scattering curves of the light state are shown with an offset on the *y* axis. (A) Shown are CRYSOL fits of the *Ms*LadC crystal structure to dark-state data in gray and the *Ms*LadC AF2 model to light-state data in cyan. (C) CRYSOL fits of the *Ao*LadC AF2 model (truncated N terminus) to either dark-state (gray) or light-state (cyan) data. Dimensionless Kratky plots of (**B**) *Ms*LadC and (**D**) NT-*Ao*LadC in dark (black) and light states (blue).

While HDX-MS highlights regions of our LadC system affected by illumination, it does not allow elucidation of a defined structure of the light-activated state, nor does it allow addressing time-resolved aspects of light-state formation. Since all attempts to crystallize *Ms*LadC and other homologs in the light-adapted conformation have failed so far, we set out to characterize the light state by solution scattering experiments. Since gel filtration experiments indicated a relatively large-scale conformational change between the dark and light states for *Ms*LadC, small-angle x-ray scattering (SAXS) should be well suited to characterize these rearrangements. To this end, we performed SAXS experiments with steady-state dark- and light-adapted *Ms*LadC. The evaluation of the solution scattering data confirmed the *Ms*LadC dimer in solution by estimating the actual molecular weight from the determined Porod volume (*V*_P_) and the volume of correlation (*V*_C_) under both steady-state non-actinic and activating conditions (table S3). However, when directly comparing the dark- and light-state data, noticeable changes between the two states become apparent (Fig. 5A and fig. S7, A and B). While the experimental scattering curve of the dark state matches the theoretical scattering profile derived from the *Ms*LadC crystal structure using CRYSOL (Fig. 5A, black and gray; χ^2^ of 2.98), the light-state *Ms*LadC features distinct features in its scattering profile (Fig. 5A, blue). There are notable differences in the radius of gyration, *R*_G_, and maximum intraparticle distance, *D*_max_, between the two states (table S3 and fig. S7, A and B). Specifically, the light-state scattering data show a larger *R*_G_ and *D*_max_ compared to the dark state. Moreover, the Kratky plot of dark-state scattering data indicates a compact and spherical shape (Fig. 5B, black), whereas the light-state data show a shift in the plot representing a less globular shape and increased degree of flexibility (Fig. 5B, blue). In this context, it is important to consider the results of the conformational dynamics experiments (HDX-MS), which suggested that different conformational states coexist in the light-activated *Ms*LadC (as evidenced by the EX1 kinetics of the linker element). Hence, the low correlation between the experimental data of the light state and the CRYSOL fit of the *Ms*LadC AF2 model (Fig. 5A; χ^2^ of 19.0) could be explained by an ensemble of conformations coexisting in the light-adapted state, especially in the absence of the substrate. This hypothesis is further supported by the light-state data’s ambiguity score of 1.56, suggesting a higher level of conformational diversity. Conversely, the dark-state scattering data have an ambiguity score of 0.30, which indicates a well-defined conformation as also supported by the Kratky plot analysis. Hence, we only calculated low-resolution envelopes of the *Ms*LadC dark state by ab initio modeling. As shown in fig. S7C, the *Ms*LadC dark-state crystal structure superposes well with the *Ms*LadC dark-state SAXS envelope (χ^2^ of 0.999), confirming the crystallographic assembly as a functionally relevant inhibited state in solution.

To further probe the concept of the inhibitory interface in the long linker homologs, we also measured solution scattering data of the short linker representative *Ao*LadC. In analogy to the similar behavior under both dark and light conditions in the gel filtration experiments, also the scattering curves of N-terminally truncated (NT-) *Ao*LadC variant remained almost identical under both conditions (Fig. 5, C and D, and fig. S7, D and E). The solution scattering data further confirmed the NT-*Ao*LadC dimer in solution (table S3). NT-*Ao*LadC displayed a distinct peak shape in the Kratky plot, featuring a characteristic shoulder that corresponds to a two-domain protein architecture (Fig. 5D). The AF2 model corresponding to the NT-*Ao*LadC sequence also produces a similarly good fit to both dark and light-state experimental scattering curves, with χ^2^ of 1.39 and 1.55, respectively (Fig. 5C), suggesting that elongated assemblies consisting of a LOV dimer flexibly tethered to a GGDEF dimer account for the observed scattering profiles under both non-actinic and activating conditions.

### Stimulating-register promoting variants

**Fig. 6.**
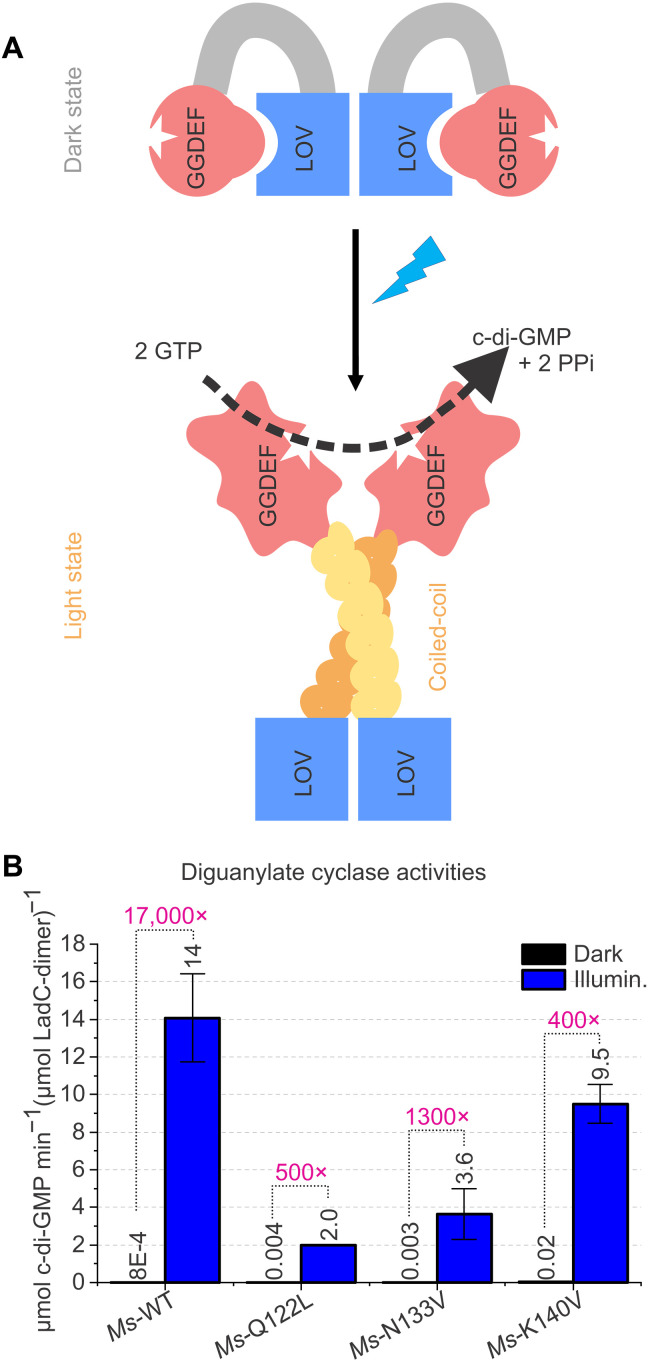
LadC switch model hypothesis. (**A**) Mechanistic model of the LadC switch, where blue-light exposure triggers rearrangements within the inhibited dimer, allowing the extension of the linker helices into a coiled-coil. Establishing the coiled-coil and the characteristic stimulating-register architecture optimally positions the GGDEF domains for efficient enzymatic function. (**B**) Enzymatic activity characterization of *Ms*LadC variants. Dark-state activities are shown in black and light-state activities in blue. The sample SD of reaction triplicates contributed to the error estimation of the linear fit used to calculate the initial rate of product formation. The SE of the linear regression estimate is shown as an error bar.

To further test the proposed two-stage activation mechanism (release of inhibitory interface and formation of a continuous coiled-coil linker; Fig. 6A), we generated amino acid substitution variants of *Ms*LadC. On the one hand, we wanted to favor the illuminated state by stabilizing the elongated coiled-coil (*17*, *31*) in its stimulating register (Fig. 3B), and on the other hand, we set out to disrupt the inhibitory interface. Hence, we prepared the Q122L, N133V, K140V, D44S/E46K, and R174S variants (fig. S8), respectively. Amino acids Q122, N133, and K140 are all part of the linker region, whereas D44 and E46 are part of the LOV core. Together with R174 from the GGDEF domain, they line the inhibitory interface between LOV and GGDEF domains. The protein variants Q122L, N133V, and K140V could be purified comparably to the wild type (WT). The double substitution D44S/E46K at the inhibitory interface could not be isolated since specific surface residues probably resulted in aggregation and irreversible binding to the Sepharose matrix during the purification. Similarly, the R174S variant showed an increased tendency for aggregation and precipitation; hence, we only focused on the linker-targeting variants. Protein variants Q122L, N133V, and K140V behaved similarly to WT in terms of spectral properties (fig. S8, A to C), with slight differences in the thermal recovery kinetics (fig. S8, D to F) and in-solution behavior during SEC (fig. S8, G to I). Variants favoring the stimulating register showed, in general, an increased dark-state activity, with the variant K140V exhibiting the highest dark-state activity, resulting in lower fold changes compared to the WT (Fig. 6B). Upon illumination, variant K140V was still similarly active as the WT, whereas for the other variants, the diguanylate cycles activities were lower than that of the WT (Fig. 6B). A central role of Gln^122^ in sensor-effector communication is apparent, as the Q122L variant exhibited impaired activities compared to those of the WT. In most other LadC homologs (fig. S1), an Arg residue is conserved at this position, which points to a role of this residue in coiled-coil specificity and positioning (*17*, *31*) or integration of the LOV activation-state signal.

## DISCUSSION

Our understanding of the molecular mechanisms of light regulation in different photoreceptors has grown substantially over the years. However, light-responsive systems with true switch-like characteristics are rather scarce (*32*, *33*) since many molecular properties, ranging from local rearrangements of side chains around the cofactors to changes in global conformational dynamics and functional properties of the output domain, influence the attainable range of activation (*34*). Here, we describe LOV-regulated diguanylate cyclases, the enzymatic activity of which can be stimulated on the order of 10,000-fold and higher. The functional implications of the crystal structure and in-solution SAXS envelopes, combined with the biochemical and spectral characterization of LadC homologs, allowed us to propose a model for the regulatory mechanism that enables the subfamily with long linkers to function as molecular switches. The longer linkers are key to the formation of an extended inhibitory interface that allows caging of the GGDEF domains under non-actinic conditions but also satisfy the length requirements for coiled-coil formation to effectively stimulate diguanylate cyclase activity upon activation. The concept of diguanylate-cyclase activation by the preceding coiled-coil architecture was initially described for a phytochrome-activated GGDEF domain based on the observation of two conditional heptad-repeat patterns in the coiled-coil linker region (*14*, *35*). The stimulating-register conformation was inspired by the highly active artificial fusion of the leucine zipper part of yeast GCN4 (general control non-depressible 4) to a GGDEF domain (*36*), and analogous register annotations have recently been described for a receiver-regulated diguanylate-cyclase system (*15*). Homologs with the shorter linker (four helical turns less) apparently do not rely on the two-stage activation mechanism and most likely cannot form the GGDEF-caged assembly, since no pronounced shift in elution volume was observed in the corresponding SEC profiles as well as no strong variation in the in-solution scattering. The absence of a tightly caged globular shape and the clear presence of a two-domain architecture in the Kratky plot analysis can be attributed to fewer degrees of freedom of the short linker compared to the long linker in LadCs. While this results in typically higher dark-state activities, moderate activation by light is still feasible due to the modulation of the relative stabilities of inhibiting and stimulating coiled-coil registers.

Previously characterized LOV-GGDEF systems originating from more complex LOV-GGDEF-EAL systems but featuring the same linker length as the short-linker LadC subfamily (21 amino acids) did not show any light-regulated c-di-GMP formation (*37*). While one construct lacks the conserved residues in the GGDEF motif and hence cannot show diguanylate cyclase activity, the other construct apparently has all the required motifs for functionality, but since it is a truncation of a more complex multidomain protein, missing parts could play a role in overall activity of the system (*37*). In these cases, it is plausible that light sensing aims at regulation of the additional EAL domain that is frequently found in tandem with GGDEF domains. Also our initial database searches revealed several closely related proteins that also harbor C-terminal EAL domains. EAL domains feature a phosphodiesterase activity, which is responsible for the degradation of c-di-GMP, and the frequent occurrence of such GGDEF/EAL pairs points at specific interactions required for localized second messenger signaling inside the cell (*38*).

In our mechanistic model (Fig. 6A), the light-induced alteration of the hydrogen bonding network around the flavin cofactor rearranges the LOV dimer architecture, destabilizing the extended caged architecture of the linker elements and the LOV-GGDEF interfaces. Eventually, this leads to the extension of the linker helices into a coiled-coil conformation required for optimal positioning and dynamics of the GGDEF domains as needed for catalysis. Thus, our model features two crucial steps of activation. First, the release of the caged GGDEF domains from the LOV core, and second, the formation of the coiled-coil linker for tuning of activity by relative stabilities of inhibiting and stimulating registers, as previously described for the related phytochrome-linked or receiver-regulated GGDEF systems (*14*, *15*). It might be interesting to further address the time scales of LOV activation and structural rearrangements of the linker by time-resolved spectroscopy and solution scattering approaches in the future.

A similar release of effector domains has been observed in other LOV-containing photoreceptors (*39*–*41*), and, especially in the case of truncated *Avena sativa* LOV2 constructs, Jα uncaging upon illumination has been adapted for many optogenetic tools (*42*). With several different molecular mechanisms at play, these studies highlight the adaptability of light signal integration in different sensor-effector combinations. The exceptionally high dynamic range of the LadC system described here is a consequence of the tight caging in the dark and the increased conformational dynamics of the stimulating coiled-coil linker architecture preceding the enzymatic output domain. Factors positively influencing the attainable fold changes include the equimolar concentrations of the actuator and effector in the covalently coupled sensor-effector system (*43*) and the requirement for at least two blue photons for full activation of the system providing more energy for conformational changes associated with modulating bound/unbound ratios of the effector domains (*44*). In this respect, one could envisage fusing (additional) output modules to the C terminus of *Ms*LadC to harness the potential of the tightly caged system and to develop optogenetic tools with attractive dynamic ranges. Considering the structures of close-to-active GGDEF architectures (*15*, *35*) and the extended LadC model generated by AF2, the C termini of the dimeric assemblies provide an ideal target for juxtaposing effector domains with minimal requirements for unstructured linkers. Along this line, the dark-adapted crystal structure of *Ms*LadC maximally separates the C termini of the two protomers and hence would effectively prevent encounters of fused effector domains in the dark. In addition, modulating c-di-GMP levels inside bacterial or even nonbacterial cells could also be an interesting optogenetic target, as shown for other light-regulated DGCs and EAL phosphodiesterases (*45*). In this context, the exceptionally high dynamic range of *Ms*LadC could provide an interesting complementary system, especially in combination with existing red-light–regulated phosphodiesterases (*46*).

Regarding other features of the crystal structure, they are in line with several previously published LOV-containing protein structures featuring a characteristic LOV dimer architecture (*21*–*23*, *40*), where the central β sheet of the LOV domain is covered by the A′α helix from the adjacent protomer at the extended dimer interface (fig. S9, A and B). In contrast to most LOV-containing proteins, the characteristic Jα helix of *Ms*LadC does not populate an extended conformation in the dark but instead forms an important part of the caged LOV-GGDEF assembly. The highly conserved flavin binding pocket (*24*–*26*) provides all the residues required for C4a-S-cysteinyl adduct formation upon blue-light activation as well as signal transduction to the central β sheet (*9*). As indicated by our analysis of changes in conformational dynamics upon illumination, the LOV dimer interface architecture appears to be affected by adduct formation. Activation affects several loop regions of the LOV domain as they might engage in new interactions. Also conformational dynamics of the A′α helix, which is positioned at the core of the LOV dimer interface, are affected by illumination. The AF2 model of *Dp*LadC that shows the stimulating coiled-coil architecture also features characteristic changes at the LOV dimer interface. As shown in fig. S9, similar structural rearrangements have also been observed for LOV domain activation, in the context of the characteristic LOV dimer structures of the short LOV-protein PpSB1 and LOV-HK from *Brucella abortus* (*21*, *23*, *47*). Eventually, these rearrangements at the LOV dimer interface are directly coupled to the linker region and together with other elements enable the release of the caged linker and the LOV-GGDEF interface. The Fα helix, for example, exhibits increased dynamics as it no longer contacts the dark-sequestered GGDEF domain in the light state. Such rearrangements, accompanied by the formation of an extended Jα (linker) architecture, are in line with the in-solution scattering data and the predicted AF2 model. As indicated by the EX1 kinetics of H/D exchange (*30*) in several linker peptides, distinct conformations exist in the light state, possibly corresponding to an equilibrium of elongated flexible versus caged architectures. Such an equilibrium provides a plausible explanation for the light-state scattering data of *Ms*LadC that do not show a clearly defined multidomain protein in the Kratky plot analysis.

The proposed active LadC conformation also agrees with the requirements for GGDEF active site formation (*12*) and the central role of the wide turn at the beginning of the GGDEF domain (*15*). Supported by the evolutionary conserved coiled-coil characteristics of the linker sequences, especially the possibility to define alternative registers with a similar coiled-coil propensity, and previous observations for related GGDEF-containing systems (*14*, *15*, *36*), diguanylate cyclase activity can be fine-tuned by the relative stabilities of stimulating and inhibiting linker architectures. Also, AF2 predictions indicate that the relative energies of the two registers are similar, as evidenced by predictions showing inhibiting- and stimulating-register conformations for the two short homologs, *Ao*LadC and *Dp*LadC, respectively (fig. S5). An Asn residue at position a* of the stimulating register, which is conserved for several of the long linker LadC homologs, was shown to promote the dynamics of the coiled coil (*17*, *31*), thereby positively affecting the stimulation of the catalytic activity in GGDEF domains (*14*). In addition, the *Ms*LadC variants targeting the linker sequence to enhance the stability of the stimulating register further support the proposed model, although the inhibitory interactions in the dark-state LOV assembly dominate the inactive state.

In summary, the sensor-effector linker composed of the typical LOV–Jα helix fused to the characteristic coiled-coil elements of the GGDEF domains preceding the DxLT wide turn motif is at the center of the regulatory mechanism. This linker provides the key regulatory element for the formation of an enzymatically competent architecture via a two-stage activation process. Such a mechanism has not been described so far, as it contains elements from release models (*39*, *48*, *49*), register-switching models (*14*, *15*), and other models with the coiled-coil linker helices at the center of regulation (*23*, *50*).

LOV-GGDEF photoreceptor systems with long sensor-effector linkers, as characterized in this work, provide an attractive way of controlling biological responses governed by c-di-GMP in bacteria with blue light. Advances in technology and a more comprehensive understanding of the molecular mechanisms could also provide a platform for new design strategies of optogenetic tools for cell biology applications that could be used in numerous biological systems. Because of extensive coevolution of the residues from both sensor, linker, and effector, applying the concept of the inhibitory interface for alternative effector fusions will not be straightforward. However, combining additional domains with inactive GGDEF constructs could be an interesting starting point for optogenetic tool development.

## MATERIALS AND METHODS

### Experimental design

Naturally occurring LadC sequences feature phylogenetic subfamilies with highly conserved differences in sensor-effector linker length. To address the influence of the linker length variation, we set out to characterize two members of each branch of the LadC family, one featuring two additional heptad repeats in the predicted coiled-coil linker.

### Protein expression and purification

Coding sequences of representative LadC proteins were extracted from National Center for Biotechnology Information BLAST and PSI-BLAST (*51*) searches (five iterations with a maximum of 1000 aligned sequences) and manually filtered by the presence of exactly one LOV and one C-terminally linked GGDEF domain. In addition, the characteristic motifs (FxxxT(G/E)Y, Gx(N/D)C(R/H)(F/I)L(Q/A), and N(Y/F)xxx(G/D)xx(F/L)xN) for the LOV domain (*13*) and DxLT and GG(D/E)EF for the GGDEF domain (*12*) were prerequisites for considering sequences as LadC homologs. Representative LadC sequences from *Methylotenera sp.*, *S. fonticola* AU-AP2C, *D. peraridilitoris* DSM 19664, and *Aquella oligotrophica* (accession nos. PPC95052.1, ERK13424.1, AFZ69583.1, and WP_102950102.1, respectively) corresponding to *Ms*LadC, *Sf*LadC, *Dp*LadC, and *Ao*LadC were codon-optimized for expression in *Escherichia coli* (GeneArt, Thermo Fisher Scientific). Gene strings were cloned into the *Nco*I- and *Not*I-linearized pET GB1a plasmid by Gibson assembly (*52*). The plasmid additionally contained the coding sequence for five Gly residues between the Tobacco Etch Virus (TEV) protease cleavage site and the start of the LadC sequence to improve protease efficiency for His-GB1 removal. For *Ao*LadC, we specifically engineered an N-terminally truncated variant (NT-*Ao*LadC) that eliminated the first 12 amino acid residues. This construct was generated due to its extended N terminus as compared to other LadC homologs (fig. S1). The absence of a structured region in this segment, as indicated by the predicted AF2 model, encouraged us to minimize the presence of unstructured regions for the solution scattering experiments. We confirmed that NT-*Ao*LadC exhibited the same in solution behavior as full-length *Ao*LadC by recovery kinetics, gel filtration, and enzyme activity assay (fig. S3A). *Ms*LadC variants and NT-*Ao*LadC were produced by one-step site-directed mutagenesis following the protocol described by Liu and Naismith (*53*) using pET GB1a *Ms-/Ao-*LadC plasmids as templates (used primers are listed in table S4).

Plasmids containing the coding sequences of the different LadC homologs with additional N-terminal TEV-cleavable 6His- and GB1-tags were transformed into RbCl-competent *E. coli* BL21 (DE3) cells. Liquid cultures in LB medium supplemented with kanamycin (0.03 g/liter) were grown at 37°C to mid-log phase (optical density at 600 ~ 0.7). After temperature reduction to 16°C, protein expression was induced with 0.1 mM isopropyl-β-d-thiogalactopyranoside, and cells were incubated for additional 16 hours at 16°C. Harvested cells (15 min, 4500 rpm, 4°C using a JLA 8.1000 rotor, Beckman Coulter) were then resuspended in lysis buffer [50 mM Hepes (pH 7.0), 500 mM NaCl, 2 mM MgCl_2_, and 30 mM imidazole] and disrupted by combined treatment with lysozyme (0.2 mg/ml) and sonification (3 × 5 min, 100 W, continuous mode, Labsonic L, Satorius). Insoluble cell materials were separated from the soluble fraction by ultracentrifugation (208,323.3*g*, 4°C, 45 min). The supernatants were affinity-purified on a Ni^2+^-Sepharose matrix (Ni Sepharose 6 Fast Flow, GE Healthcare) using a gravity-flow protocol in Protino columns (Macherey-Nagel). After loading, the matrix was washed with three column volumes of lysis buffer supplemented with 100 μM FMN (Panreac, AppliChem) and then with six column volumes of lysis buffer containing 40 mM imidazole. The proteins were eluted with four column volumes of lysis buffer containing 250 mM imidazole. The N-terminal 6His-GB1-Tag was removed by in-house expressed His-tagged TEV protease at a ratio of 1:10 (TEV/substrate) during overnight dialysis at 4°C in dialysis buffer in which the imidazole concentration was reduced to 45 mM. LadC homologs featuring longer linkers were more sensitive toward high salt concentrations. For this reason, from dialysis onward, different buffer compositions were used for the two subfamilies, as shown in table S5. The cleaved protein solution was reloaded onto the Ni^2+^ column to remove contaminants that bind unspecifically to the Ni-NTA material and the TEV protease, and LadC homologs were collected in the flow through. The samples were concentrated using centrifugal filters (Ultracel-10K, Merck Millipore) and further purified by SEC on a Superdex 200 Increase 10/300 GL column (GE Healthcare) equilibrated in storage buffer (table S5). Purified proteins were flash-frozen in liquid nitrogen and stored at −80°C until needed.

### Spectroscopic and kinetic characterization

Ultraviolet-visible (UV-Vis) absorption spectra were measured with a UV-Vis Specord 200 PLUS spectrophotometer (Analytik Jena) in the storage buffer (table S5). Measurements were performed at 20°C. The dark state was previously equilibrated under non-actinic conditions, and the light state was measured immediately after blue light illumination [Spot light-emitting diode (LED) with 48 LEDs SMD, 1.2 mW cm^−2^, 465 nm, Luminea]. The recovery kinetics were followed by measuring the absorbance at 448 nm instantly after illumination with blue light in a storage buffer and at 20°C.

The conversion of GTP to c-di-GMP or pppGpG was measured by high-performance liquid chromatography (HPLC), as previously described by Böhm *et al.* (*46*), with an adapted protocol. For initial scouting experiments, we varied reaction conditions as summarized in fig. S3. The protein concentration of each homolog was adjusted so that the specific activity reached a plateau when plotted as a function of concentration. The time points were adjusted so that the conversion of substrate did not exceed 10%. Proteins (2 μM) were mixed with GTP (50 μM, GE Healthcare) in reaction buffer (table S5), except for *Sf*LadC, which was measured at 1 μM protein due to very high turnover and limited time frame of handling. Protein dilutions were equilibrated at 20°C. Reactions were started by addition of GTP. The dark-state reactions were incubated under non-actinic conditions, and light-state reactions were incubated under blue light (Mounted LED, 455 nm, Thorlabs, 1.5 mW cm^−2^) during the whole measurement following a 1-min pre-illumination step. The reactions were stopped at different time points by denaturation at 95°C for 1 min and centrifuged before loading onto the HPLC column. For *Ao*LadC and *Dp*LadC, the reactions were additionally quenched with 35 mM EDTA before denaturation due to high conversion at elevated temperatures (fig. S3). The supernatants were analyzed using a reversed-phase column ProntoSIL 120-3-C18 ACE-EPS 4.6 mm by 100 mm, Bischoff, equilibrated in 10 mM potassium phosphate buffer (pH 8.0) and 6% methanol. The separation was performed with a gradient to 10% methanol over 3 min and reequilibration with 6% methanol over 6 min at a flow rate of 1 ml/min (fig. S3I). Absolute c-di-GMP amounts were quantified by the relative amount of product being formed with respect to the known GTP concentration. All enzymatic activities were normalized to the concentration of the dimeric LOV-GGDEF homolog.

### SEC/MALS

Between 500 and 1000 μg of protein were analyzed for each LadC homolog in the dark-adapted and the illuminated state using a Superdex 200 Increase 10/300 GL column. The protein samples were centrifuged (16,000*g*, 5 min) before loading, and the column was equilibrated in storage buffer. When analyzing the light state, the sample was illuminated with blue light (Spot LED with 48 LEDs SMD, 1.2 mW cm^−2^, 465 nm, Luminea) for 1 min and then loaded onto the column, which was illuminated from both sides during the whole run with blue light. All SEC runs were carried out on SEC columns of the same dimensions but partially on different chromatographic systems. To determine the molar mass of the eluting species, the SEC column was coupled to a MALS detector (miniDAWN, Wyatt Technologies). Protein concentrations were estimated on the basis of the absorbance at 280 nm and the calculated extinction coefficients of the respective protein samples, also accounting for the flavin contribution at this wavelength (~19,000 M^−1^ cm^−1^). Data were evaluated with the ASTRA software package (Wyatt Technologies).

### Crystallization and structure elucidation

Dark-adapted *Ms*LadC was crystallized at 20°C in a sitting-drop vapor diffusion setup using an Oryx 8 crystallization robot (Douglas Instruments). Drops (1 μl) containing equal volumes of protein (1.5 mg/ml) and reservoir solution (table S6) and 0.1 μl of seeds from previous crystallization setups, which were grown in storage buffer. In general, *Ms*LadC showed a propensity for crystallizing even in the storage buffer, and at elevated concentrations, microcrystals were most likely formed. The seeds were prepared by crushing crystals grown in storage buffer. Crystallization plates were incubated under non-actinic conditions, and bipyramid-like crystals appeared after overnight incubation, reaching final dimensions within 1 week. Harvested crystals were flash-frozen and stored in liquid nitrogen, 77 K. Diffraction data were collected at beamline P11 at PETRA III, DESY (*54*, *55*). Native datasets were collected from one crystal (*Ms*nat.1 and *Ms*nat.2; table S2) and SAD datasets from another crystal (*Ms*SAD.1, *Ms*SAD.2, and *Ms*SAD.3; table S2). The crystal structure of *Ms*LadC was solved by a combination of molecular replacement (MR) and sulfur single-wavelength anomalous dispersion (S-SAD) phasing using an adapted refinement protocol due to atypical intensity distributions of the observed reflections (Wilson distribution in fig. S4B). The diffraction data were integrated and scaled using the XDS package (*56*). In addition, datasets from identical crystals were merged with XSCALE. For molecular replacement, one LOV domain and one GGDEF domain of the *Ms*LadC AF2 model, both with truncated loop regions, and missing the cofactor, the A′α helix as well as the sensor-effector linker helix, were successfully placed in the asymmetric unit of the *Ms*nat. merged dataset using PHENIX Phaser (*57*, *58*). Missing regions (A′α helix, sensor-effector linker, and loop architectures) were manually built in the Phaser density map, and the resulting model was refined using PHENIX Refine (*59*, *60*). Subsequent rounds of refinement consisted of placing real space-refined models [based on σA-weighted 2mF_O_-DF_C_ and F_O_-F_C_ electron density maps of Refine runs in combination with initial unweighted Phaser density maps in WinCoot (*61*)] using PHENIX Phaser followed by another cycle of PHENIX Refine. This unusual structure refinement strategy was used since consecutive maximum likelihood refinement runs in Refine could not properly deal with the atypical intensity distribution of the reflections, which resulted in a loss of electron density for some side-chain features that were clearly visible already in the initial Phaser run. We attribute difficulties in structure solution and still rather high R values to an unexplained pathology of the crystals, since attempts to account for pathologies, such as twinning and testing several different crystals, were unsuccessful. The *Ms*nat. model was then used as a search model for Phaser runs of the *Ms*SAD merged dataset, and anomalous data were obtained using PHENIX AutoSol (*62*) based on the initial MR solution. The resulting model was similarly manually refined through several rounds of Phaser and Refine using experimental phases from AutoSol as additional restraint. This model was eventually used for phasing the higher resolution *Ms*nat. merged dataset. Refinement included initial rigid body fit, six TLS groups, secondary structure restraints, and a final optimization of x-ray/stereochemistry and x-ray/atomic displacement parameter weights using Refine. To assess our strategy, we checked for model bias effects by deleting larger parts of the model, as well as deleting single side chains, and verifying the presence of electron density directly after the Phaser run, in addition to checking the quality of the electron density of the flavin cofactor (fig. S4C).

### AF2 structure prediction

Structure models for *Ms*LadC, *Sf*LadC, *Ao*LadC, and *Dp*LadC were generated by AF2 (*63*) via AlphaFold-multimer ColabFold. (*64*). The number of recycles was set as 15, with homo-oligomers (the “Supplementary Code” section in the Supplementary Materials).

### HDX-MS

The *Ms*LadC stock solution (61 μM) was aliquoted (4.5 μl) in reaction tubes and equilibrated under non-actinic light (dim red or orange light) conditions at 20°C for 1 min. For analyzing the illuminated state, samples were pre-irradiated with blue light (Mounted LED, 455 nm, Thorlabs, 1.5 mW cm^−2^) for 1 min at 20°C. Deuterium exchange reactions were started with a 1:15 dilution in deuterated buffer [10 mM Hepes (pD 7.0), 50 mM NaCl, and 2 mM MgCl_2_]. Transparent reaction tubes were kept closed to prevent condensation of water in the tubes during longer incubation times. Aliquots (10 μl) were removed and quenched at 10 s, 45 s, 3 min, 15 min, and 60 min with 10-μl ice-cold 200 mM ammonium formic acid (pH 2.5), 1.5 M urea and flash-frozen in liquid nitrogen. Before injection into the HPLC system, aliquots were thawed by resuspension in ice cold quenching buffer in a total volume of 55 μl. Samples of 50 μl were then injected into a cooled HPLC setup, as described previously (*35*). Briefly, protease digestion was performed on an immobilized pepsin column (BEH Enzymate, Waters) operated at 10°C and 0.3 ml/min. Peptide fragments were then desalted on a C18 trap column [Shim-pack GISS-HP(G), Shimadzu] and separated during a 4.25-min acetonitrile gradient (10 to 45%) in the presence of 0.6% (v/v) formic acid on a reversed-phase column (Shim-pack Arata Peptide, Shimadzu). Separated fragments were then infused into an impact II ESI-Q-TOF (Bruker). Deuterium incorporation was analyzed and quantified with the Hexicon2 software package (*65*). Data are summarized in table S7.

### In-solution scattering analysis

*Ms*LadC (9.4 mg/ml) was loaded onto a Superdex 200 Increase 10/300 GL column equilibrated in storage buffer right before measurement on BM29 at the ESRF (Grenoble, France) (*66*). Collected fractions were kept in dark at 4°C. NT-*Ao*LadC was thawed and centrifuged before measurement. The x-ray wavelength was 0.99 Å. The temperature in the sample holder was 4°C and in the capillary during SAXS measurement 20°C. The *Ms*LadC protein concentration was 0.8 mg/ml, and NT-*Ao*LadC was 0.9 mg/ml. Exposure time was 1 s per collected frame, and 10 frames were recorded per 50 μl sample per condition. Dark-state samples were prepared under orange light conditions, and for the measurement, the cold light source filter next to the sample capillary was switched to red. The light-state sample was illuminated with blue light (Spot LED with 48 LEDs SMD, 1.2 mW cm^−2^, 465 nm, Luminea) before loading into the sample holder, where illumination continued from the top after hutch closing including the transparent tube preceding the capillary. For the measurement, the cold light source filter was switched to blue, intensity setting at 80%. Data were analyzed with RAW software package version 2.1.2 (*67*), ambiguity scores were calculated from P(r)-functions using ATSAS AMBIMETER (*68*), and ab initio low-resolution envelope was generated by GASBORp (*69*) (table S3). Theoretical scattering curves were calculated and fitted to experimental data using ATSAS CRYSOL (*70*).

### Statistical analysis

For the enzymatic activity assay, each time point was prepared in triplicate. Sample SDs were then calculated for each point and contributed to the error estimation of the linear fit that was performed using Origin 2019.

For HDX-MS experiments, each time point was prepared in biological triplicates. Sample SDs were then calculated using Hexicon2 for the centroids of each peptide’s isotope pattern.

## Supplementary Materials

### This PDF file includes:

Figs. S1 to S9 Tables S1 to S7 Legend for Supplementary code Legend for Supplementary HXD data

### Other Supplementary Material for this manuscript includes the following:

Supplementary code

Supplementary HXD data
