# Supplementary material for: Illuminating the inner workings of a natural protein switch: Blue-light sensing in LOV-activated diguanylate cyclases
Source: Sci Adv. 2023 Aug 2;9(31):eadh4721. doi: 10.1126/sciadv.adh4721 (PMC10396304; doi:10.1126/sciadv.adh4721)
Supplement: Supplementary file 1 — Figs. S1 to S9 Tables S1 to S7 Legend for Supplementary code Legend for Supplementary HXD data [file sciadv.adh4721_sm.pdf]

Science Advances
AAAS

Supplementary Materials for

Illuminating the inner workings of a natural protein switch: Blue-light sensing in LOV-activated diguanylate cyclases

Uršula Vide *et al.*

Corresponding author: Andreas Winkler, andreas.winkler@tugraz.at

Sci. Adv. **9**, eadh4721 (2023)
DOI: 10.1126/sciadv.adh4721

The PDF file includes:

Figs. S1 to S9
Tables S1 to S7
Legend for Supplementary code
Legend for Supplementary HXD data

Other Supplementary Material for this manuscript includes the following:

Supplementary code
Supplementary HXD data

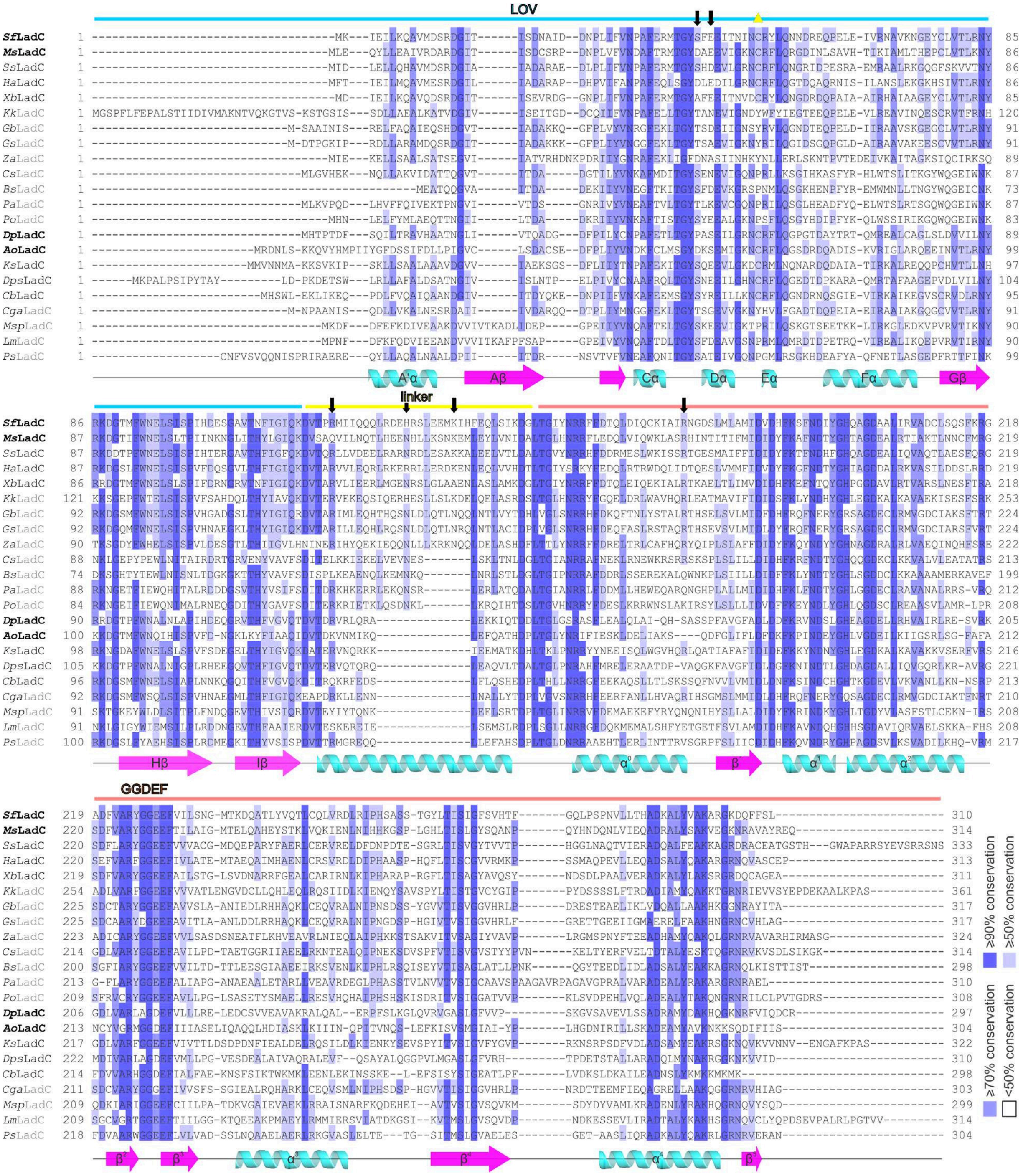

Fig. S1.

Alignment of homologous LOV-GGDEF sequences. Sequences containing the typical motifs for LOV and GGDEF domains, as well as the Cys residue (highlighted by yellow triangle) essential for the photoactivation are indicated by LadC written in black. Sequences missing either the adduct-forming Cys residues and/or other typical motifs of the LOV domain are indicated by LadC written in gray. The color scheme corresponds to the degree of conservation.

Sequences are grouped based on their linker length that differs by multiples of 7. LadC homologs characterized in this work are highlighted in bold. Black arrows indicate the positions of *Ms*LadC variants. The sequences were aligned by ClustalO and corresponding accession numbers are: *Sf*LadC ERK13424.1, *Ms*LadC PPC95052.1, *Ss*LadC ERJ18220.1, *Ha*LadC CAL62549.1, *Xb*LadC PZO09896.1, *Kk*LadC WP_015780739.1, *Gb*LadC OGT00165.1, *Gs*LadC TAN75059.1, *Za*LadC WP_008253335.1, *Cs*LadC QOR66207.1, *Bs*LadC WP_120032414.1, *Pa*LadC WP_134756714.1, *Po*LadC WP_190931151.1, *Dp*LadC AFZ69583.1, *Ao*LadC WP_102950102.1, *Ks*LadC AOE49495.1, *Dps*LadC WP_124873367.1, *Cb*LadC PIS00708.1, *Cga*LadC KXS31635.1, *Msp*LadC WP_009832082.1, *Lm*LadC WP_022953060.1, *Ps*LadC WP_122223750.1. Secondary structure elements placed below the alignment are based on LOV and GGDEF architectures *(12, 24)* and the Jpred tool (JalView).

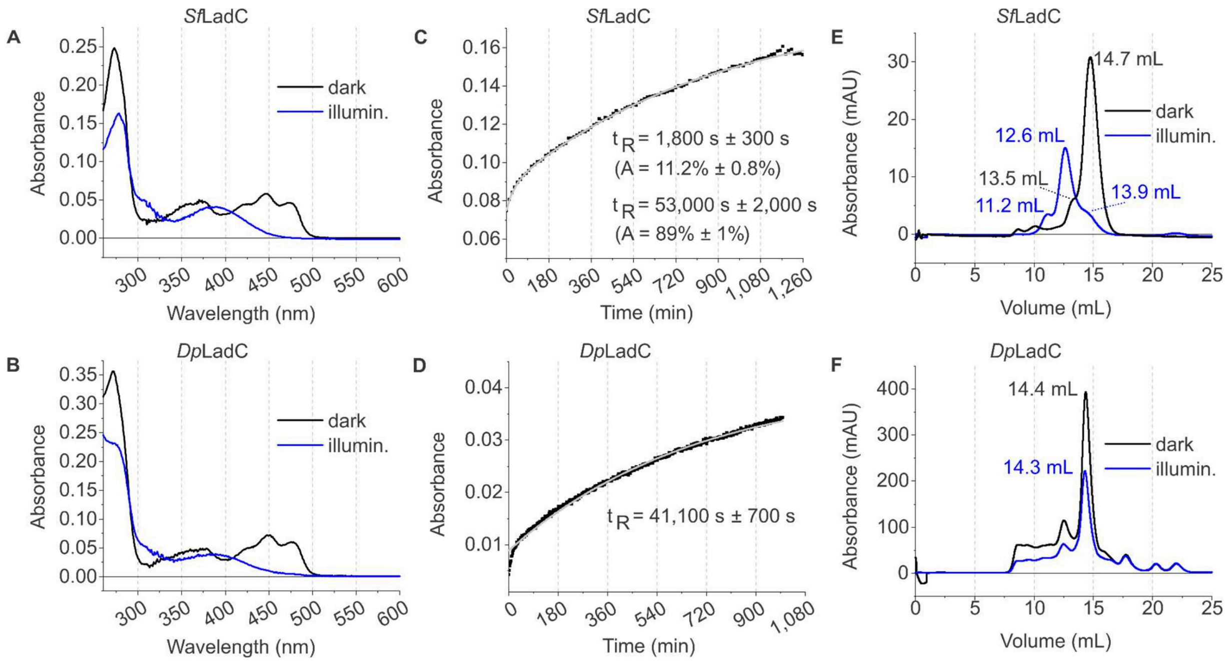

Fig. S2.

Biochemical characterization of LadC homologs. **A,B**) UV-visible spectra of *Sf*LadC and *Dp*LadC. The spectra show characteristic absorption of protein bound oxidized flavin (dark, black) and the flavin C4a-cysteinyl adduct species (illuminated, blue) following the excitation with blue light; **C,D**) Dark-state recovery kinetics of *Sf*LadC and *Dp*LadC. Interestingly, *Sf*LadC's thermal recovery displayed a two-phasic trend (2nd order exponential decay function fit in grey). Based on each oligomer's peak fraction in SEC profile (**e**) and the amplitudes of recovery phase, a possible explanation for two-phasic thermal recovery could be that dimers recover faster than tetramers or higher oligomeric species; **E,F**) Gel-filtration-chromatograms show in-solution light-induced structural changes of *Sf*LadC and *Dp*LadC. Shown are 280 nm-absorbance traces.

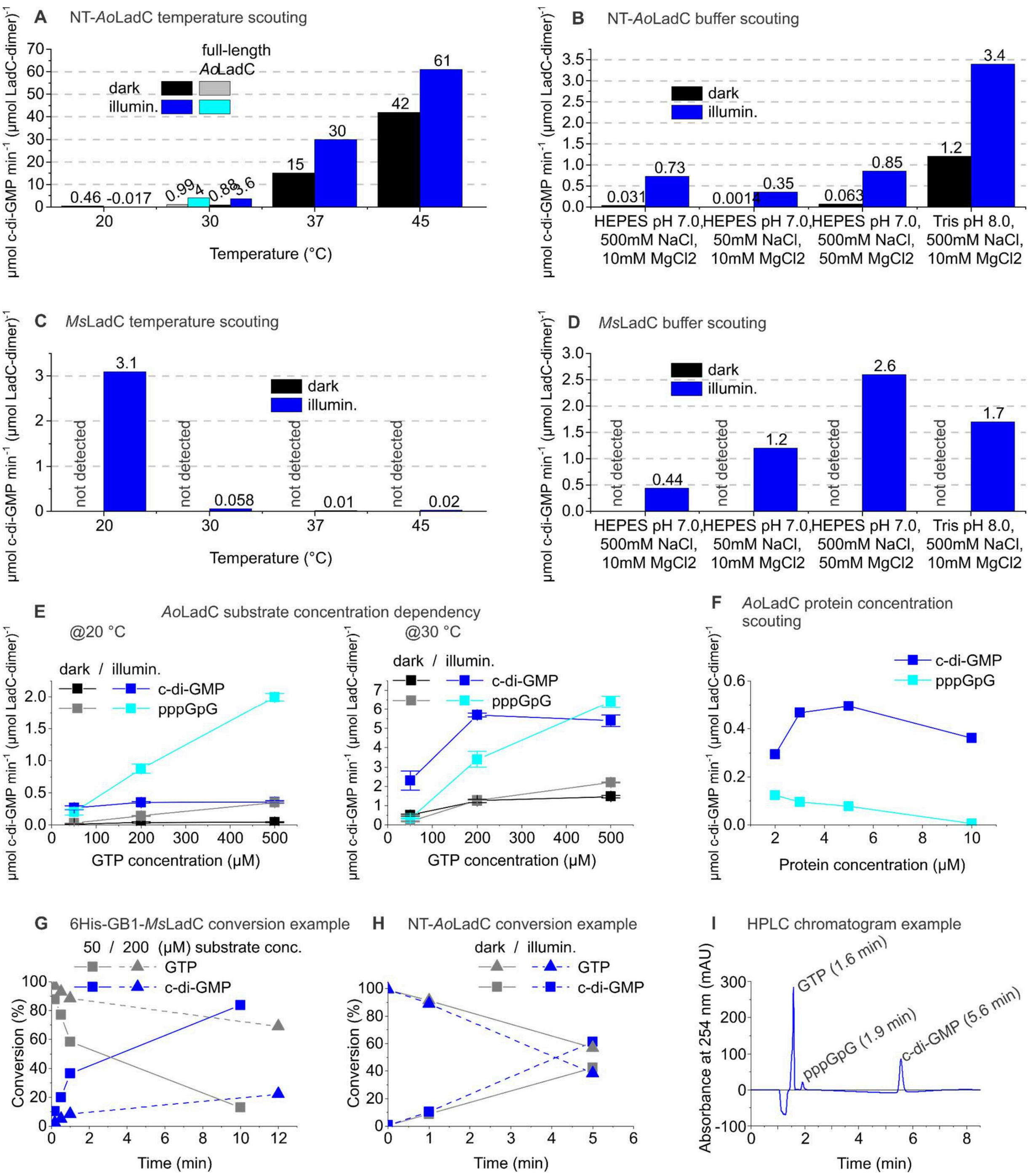

Fig. S3.

Scouting experiments for enzyme kinetics characterization of LadC homologs.

A) Temperature dependency and **B**) buffer composition scouting of the short-linker representative NT-*Ao*LadC. Full length *Ao*LadC exhibits the same behavior as shown in panel **A** at 30 °C. The NT-*Ao*LadC temperature dependency was measured at 0.2 µM protein and 100 µM GTP, in 50 mM Tris, pH 8.0, 500 mM NaCl, 10 mM $MgCl_2$. Buffer scouting was measured with 10 µM protein and 100 µM GTP, at 20 °C; **C**) Temperature dependency and **D**) buffer

composition scouting of the long-linker representative *Ms*LadC. For *Ms*LadC the temperature dependency was measured with 0.4 µM protein and 100 µM GTP, in 50 mM Tris, pH 8.0, 500 mM NaCl, 10 mM $MgCl_2$. Buffer scouting was measured with 0.8 µM protein and 100 µM GTP, at 20 °C; **E**) Specific activities of *Ao*LadC's enzymatic activity plotted against substrate concentration, measured either at 20 or 30 °C. Shown are activities for the formation of c-di-GMP and pppGpG. Activities at 20 °C were measured as described in the Methods section, activities at 30 °C were measured at 0.3 µM protein in the same buffer as described in Methods section; **F**) Testing the influence of *Ao*LadC protein concentration on pppGpG formation. Activities were measured at 20 °C in the same buffer as described in the Methods section; **A-F**) Sample standard deviations of triplicates measurements contributed to the error estimation of the linear fit used to calculate the initial rate of product formation. The standard error of the linear regression estimate is shown as an error bar. Values with no error indicator were measured without replicates during the initial scouting experiments as initial reference points for obtaining suitable reaction conditions for detailed analyses; **G**,**H**) Examples of GTP conversion progress curves of the two linker cluster representatives, *Ms*LadC (in the presence of solubility tag) and NT-*Ao*LadC, respectively. *Ms*LadC was measured at 2 µM protein in same reaction conditions as described in the Methods section. NT-*Ao*LadC was measured at 0.2 µM protein and 100 µM GTP, at 45 °C in 50 mM Tris, pH 8.0, 500 mM NaCl, 10 mM $MgCl_2$; **I**) Detection of substrate, intermediate and product as performed using the RP-HPLC protocol described in the Methods section. Shown is an example chromatogram trace at 254 nm.

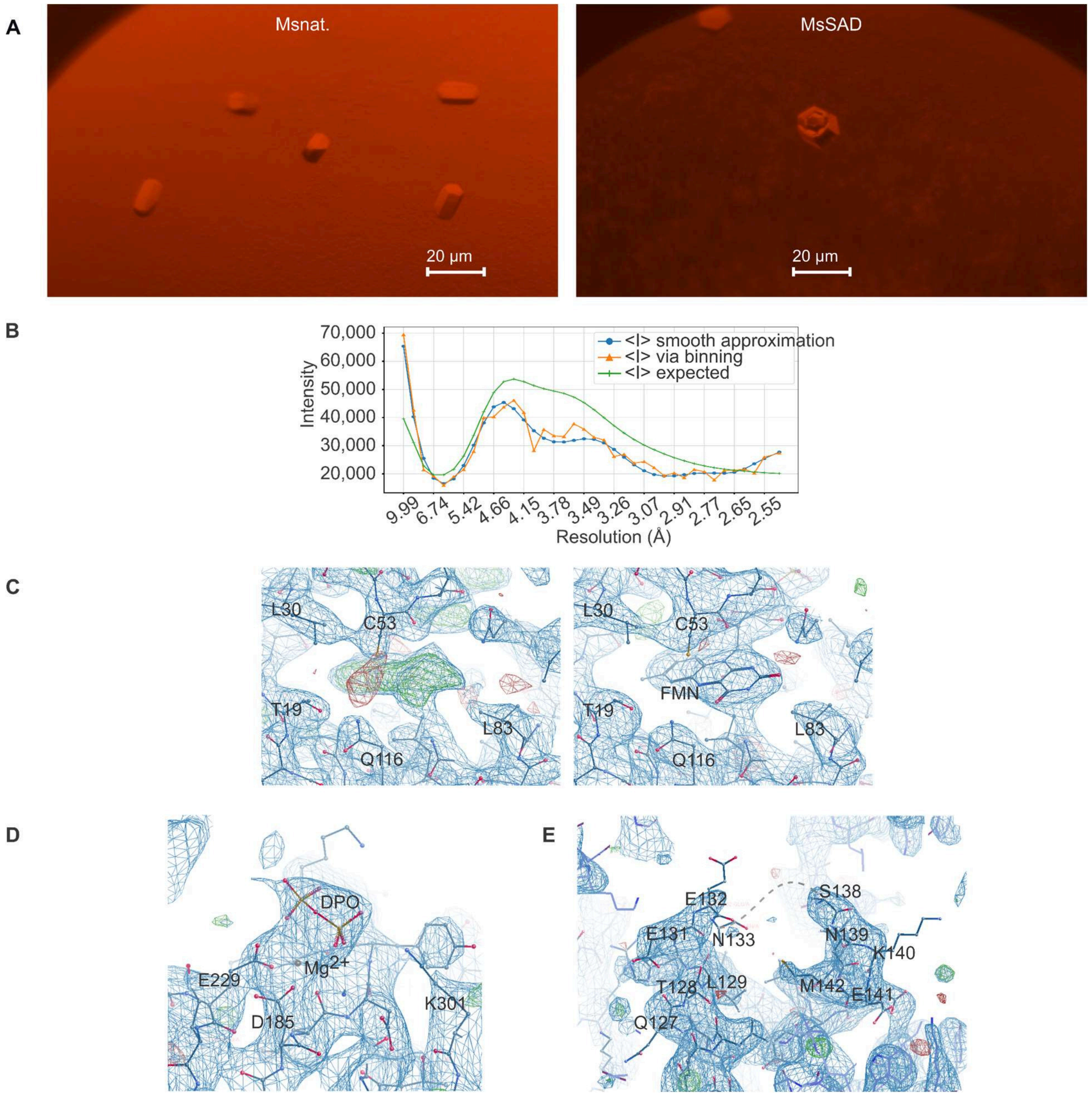

Fig. S4.

Crystal structure of full-length dark state *Ms*LadC. **A**) Representative protein crystals that were used for data collection at the synchrotron; **B**) Wilson distribution of the observed reflections, generated by Xtriage from the Phenix package; **C**) Close-up view of the flavin binding pocket. The left panel shows the electron density with the corresponding model directly after MR using Phaser. The right panel shows the final refined electron density with the corresponding model; **D**) Close-up view on the pyrophosphate binding site with a Mg^{2+} ion; **E**) Close-up view of the linker region, where four disordered residues between Asn133 and Ser138 were not built into the model. Shown are snapshots from Coot with a contour level at 1 σ.

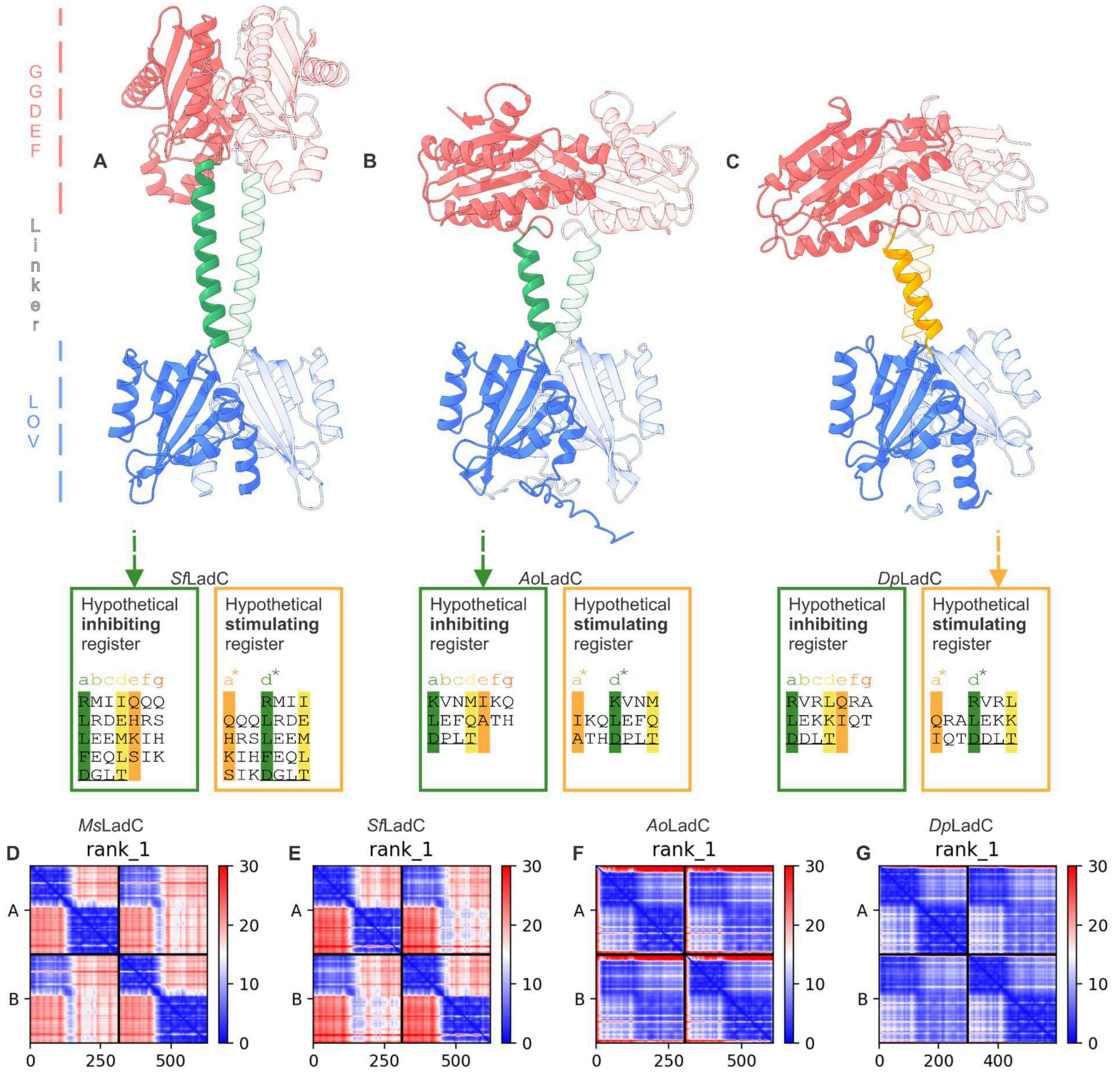

Fig. S5.

LadC structure predictions by AlphaFold2 and corresponding hypothetical linker registers. **A**) *Sf*LadC and **B**) *Ao*LadC linker residues in the AlphaFold2 model correspond to the hypothetical inhibiting register; **C**) *Dp*LadC linker residues in the AlphaFold2 model align with the hypothetical stimulating register. The underlined aa residues correspond to the wide turn region at the N-terminal end of the GGDEF domain; **D-G**) Corresponding PAE plots of the LadC models in main text Fig. 3 and panels A to C of this figure, respectively.

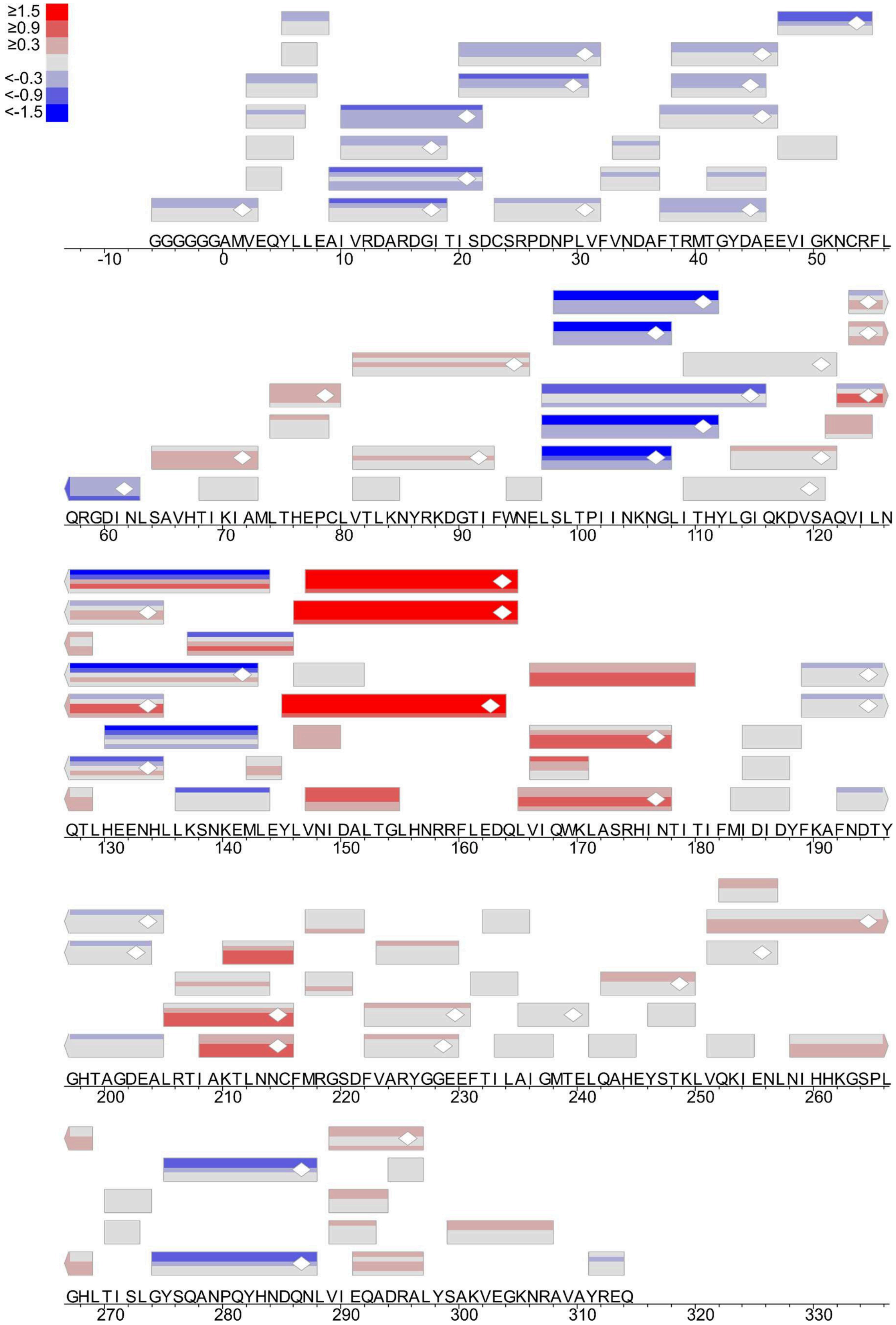
≥1.5
≥0.9
≥0.3
<-0.3
<-0.9
<-1.5
GGGGGGAMVEQYLLEAI VRDARDGI TI SDCSRPDNPLVFVNDAFTRMTGYDAEEVI GKNCRFL
-10 0 10 20 30 40 50
QRGDI NLSAVHTI KI AMLTHEPCLVTLKNYRKDGTI FWNELSLTPI I NKNGLI THYLGI QKDVSAQVI LN
60 70 80 90 100 110 120
QTLHEENHLLKSNKEMLEYLVNI DALTGLHNRRFLEDQLVI QWKLASRHI NTI TI FMI DI DYFKAFNDTY
130 140 150 160 170 180 190
GHTAGDEALRTI AKTLNNCFMRGSDFVARYGGEEFTI LAI GMTELQAHEYSTKLVQKI ENLNI HHKGSPL
200 210 220 230 240 250 260
GHLTI SLGYSQANPQYHNDQNLVI EQADRALYSAKVEGKNRAVAYREQ
270 280 290 300 310 320 330

Fig. S6.

HDX-MS characterization of *Ms*LadC. Shown is the peptide map of full length *Ms*LadC. Analyzed peptides are colored according to differences in deuterium uptake (ΔDrel) at various time points of deuteration increasing from bottom to top. Diamond symbols on a peptide indicate its MS^2 sequence confirmation.

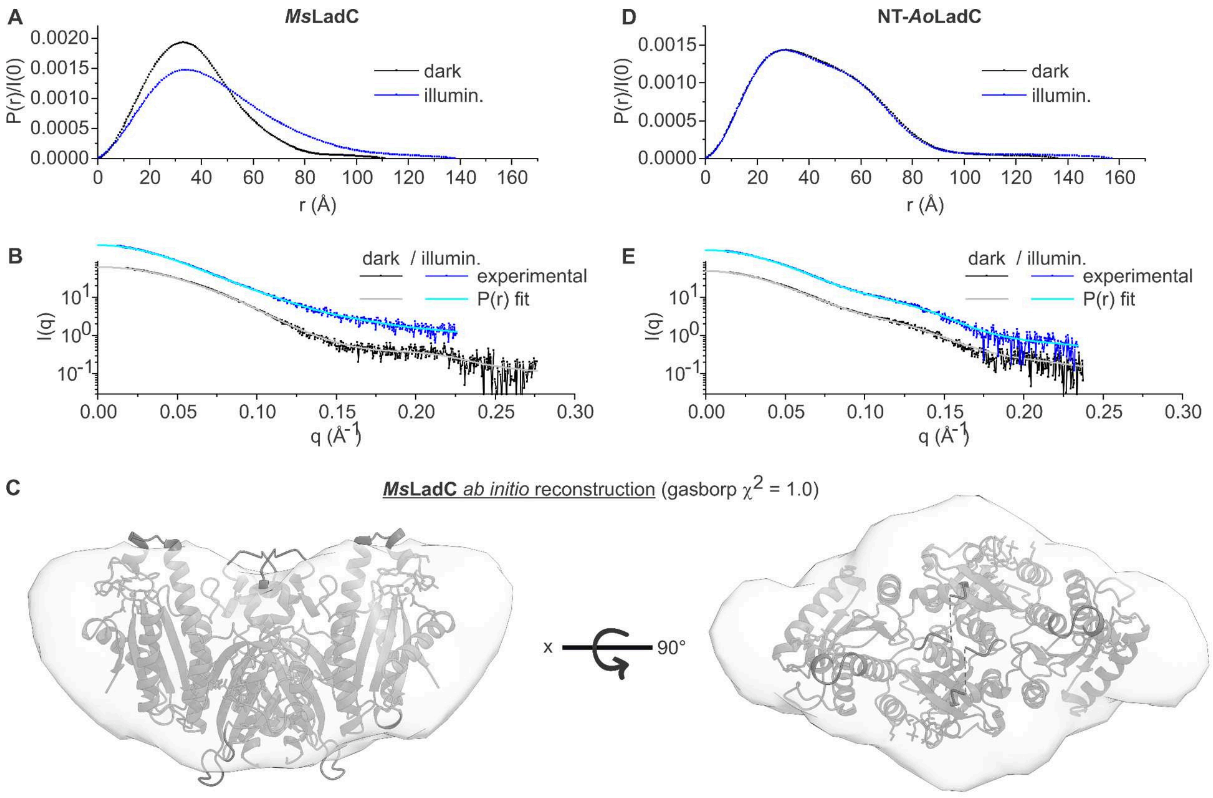

Fig. S7.

SAXS plots of *Ms*LadC and NT-*Ao*LadC in dark- (black) and light-state (blue). **A**,**D**) Distance distribution functions P(r) and the corresponding **B**,**E**) function fits. Scattering curves of the light state are shown with offset on the y-axis; **C**) *Ab initio* shape reconstructions of *Ms*LadC in the dark state (grey surface with transparency, SASDSC5). Crystal structure of *Ms*LadC is shown as dark grey cartoon and is superimposed with the dark-state envelope (**C**).

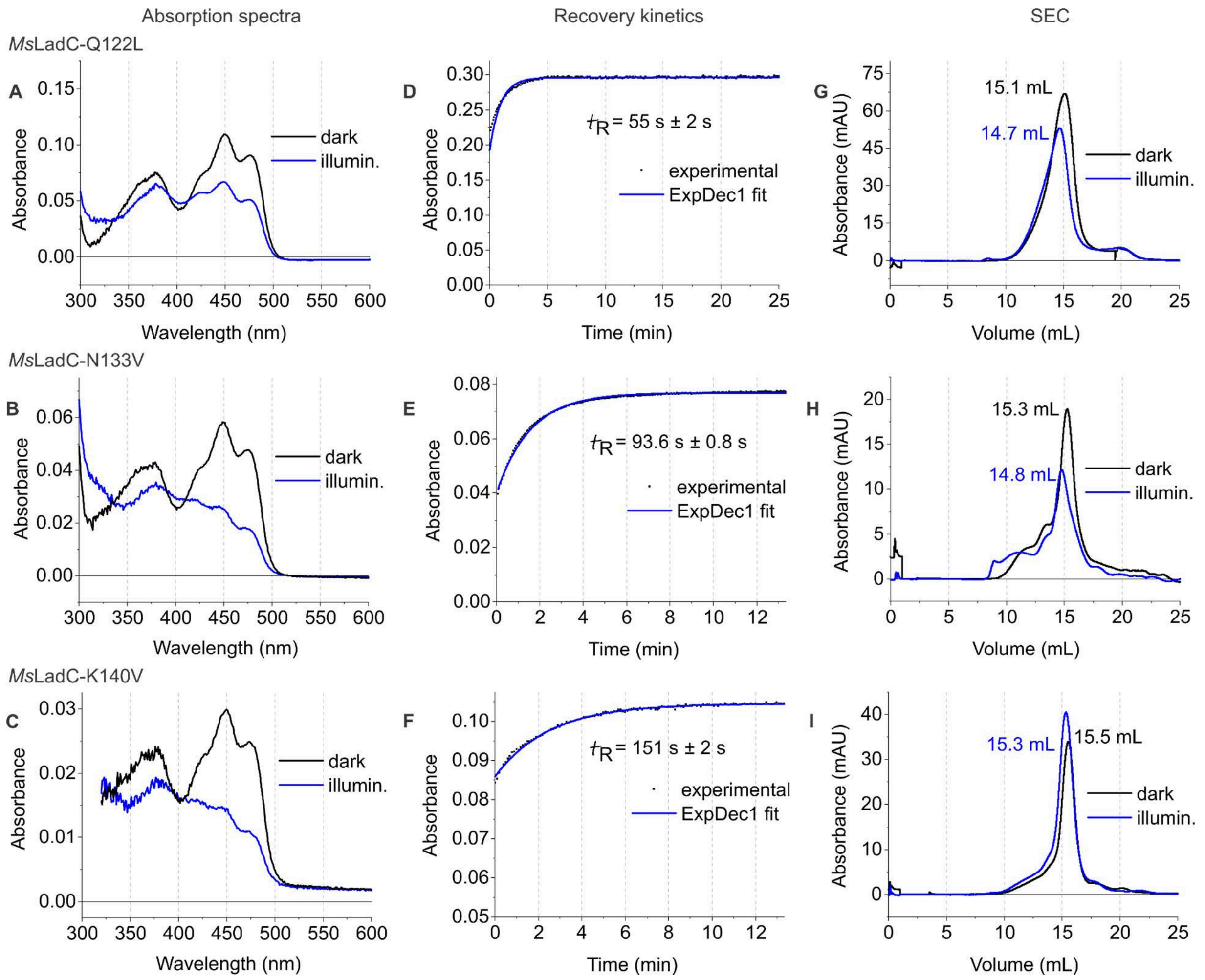

Fig. S8.

Biochemical characterization of *Ms*LadC-variants. **A-C**) UV-visible spectra of *Ms*LadC-Q122L, -N133V and -K140V. The spectra show same characteristic absorption behavior as the *Ms*LadC wild type; **D-F**) Dark-state recovery kinetics of *Ms*LadC-variants. All but *Ms*LadC-K140V show slightly faster thermal recovery as the *Ms*LadC wild type; **G-I**) Gel-filtration-chromatograms of the linker-targeting variants. Shown are 280 nm-absorbance traces. Exact elution volumes between these traces and the Fig. 1 cannot be compared because separations were run on different systems.

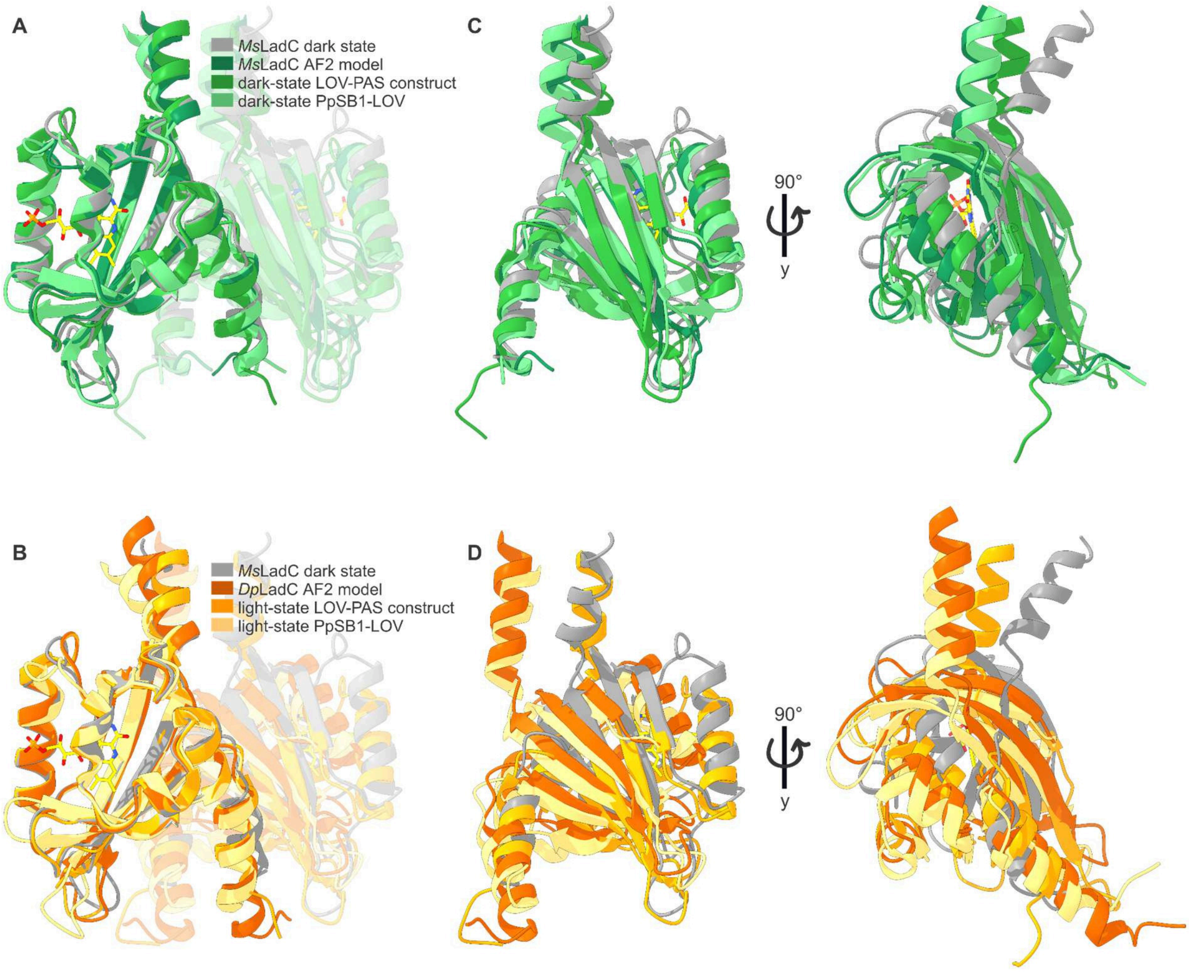

Fig. S9.

Superposition of LOV dimers with Jα onset. Protomers A of dimeric LOV assemblies were superposed based on the LOV core region and are shown in **A**) and **B**) with the respective protomer B in transparency. For clarity, protomers B are shown in an identical orientation in panels **C**) and **D**), respectively; **A,C**) *Ms*LadC AF2 model, dark-state LOV-PAS construct from LOV-HK (PDB 6PH3) and dark-state PpSB1-LOV (PDB 5J3W) superposed to chain A of the dark-state *Ms*LadC LOV dimer (grey cartoon, FMN is shown in yellow sticks) with root mean square deviations (RMSD) for main chain atoms of 0.68, 0.73 and 0.58 Å, respectively; **B,D**) *Dp*LadC AF2 model, light-state LOV-PAS construct from LOV-HK (PDB 6PPS) and light-state PpSB1-LOV (PDB 3SW1) superposed to chain A of the dark-state *Ms*LadC LOV dimer with RMSD values for main chain atoms of 0.59, 0.72 and 0.78 Å, respectively;

Table S1.

In-solution determination of *Ao*LadC and *Ms*LadC molecular weights using SEC-MALS. The values were obtained from the ASTRA® software package, Wyatt Tech.

	Peak limits (mL)	Light Scattering Model	Fit Degree	dn/dc ($^{mL}/_{g}$)	Injected Mass (µg)	Calculated Mass (µg)	Mass Recovery (%)	Molar mass ($^{g}/_{mol}$)	Polydispersity Mw/Mn
*Ao*LadC dark	13.207 – 14.767	Zimm	1	0.1850	550.00	426.69	77.6	**6.934×10^4 (±2.8%)**	1.001 (±3.9%)
*Ms*LadC dark	13.893 – 14.860	Zimm	1	0.1850	955.80	726.85	76.0	**7.389×10^4 (±4.5%)**	1.003 (±6.4%)
*Ms*LadC illumin.	13.052 – 14.169	Zimm	1	0.1850	660.80	450.88	68.2	**7.691×10^4 (±6.8%)**	1.002 (±9.6%)

Table S2.

Data collection and refinement statistics for the *Ms*LadC structure elucidation. Values in parentheses are for the highest-resolution shell.

	**Data collection**						
	***Ms*nat.1**	***Ms*nat.2**	***Ms*nat. merged**	***Ms*SAD.1**	***Ms*SAD.2**	***Ms*SAD.3**	***Ms*SAD merged**
Crystal to detector distance (mm)	740.500	303.100		183.500	183.500	183.500	
Space group	P6(1)22	P6(1)22		P6(1)22	P6(1)22	P6(1)22	
Cell dimensions<br>a b c (Å)	56.347<br>56.347<br>318.037	56.362<br>56.362<br>318.228		57.124<br>57.124<br>319.674	57.211<br>57.211<br>319.779	57.333<br>57.333<br>320.248	
α β γ (°)	90<br>90<br>120	90<br>90<br>120		90<br>90<br>120	90<br>90<br>120	90<br>90<br>120	
Wavelength (Å)	1.03312	1.03312		2.06614	2.06614	2.06614	
Resolution (Å)	50 – 3.5<br>(3.7 – 3.5)	50 – 2.5<br>(2.6 – 2.5)	50 – 2.5<br>(2.6 – 2.5)	50 – 2.8<br>(2.9 – 2.8)	50 – 2.8<br>(2.9 – 2.8)	50 – 2.8<br>(2.9 – 2.8)	50 – 2.8<br>(2.9 – 2.8)
R_{obs} (%)	4.3 (6.4)	6.7 (80.5)	5.6 (79.8)	8.9 (106)	8.1 (95.5)	14.3 (253.1)	11.9 (157.2)
I/σ(I)	35.5 (7.0)	25.9 (4.4)	27.2 (4.4)	25.4 (3.0)	29.2 (3.4)	25.6 (1.9)	41.0 (4.8)
$CC_{1/2}$ (%)	100 (99.8)	99.9 (99.2)	100 (99.3)	100 (98.8)	100 (99.3)	100 (96)	100 (99.6)
Completeness (%)	80.6 (41.2)	99.9 (99.8)	99.9 (99.8)	93.1 (97.5)	93.4 (100)	93.4 (100)	95.1 (100)
Redundancy	12.3 (2.2)	18.4 (18.6)	22.2 (18.7)	30.7 (22.7)	30.8 (22.8)	43.8 (3.5)	103.4 (79.7)

	**Refinement**	
	***Ms*nat.merged**	***Ms*SADmerged**
Resolution (Å)	48.801 - 2.500 (2.589 - 2.500)	49.467 - 2.800 (2.900 - 2.800)
No. reflections	11,153 (1,063)	7988 (818)
R_{work} / R_{free}	0.2547 (0.3316) / 0.2978 (0.3971)	0.3126 (0.3775) / 0.3381 (0.4065)
B factor average	80.05	82.86
RMSD<br>Bond lengths (Å)<br>Bond angles (°)	 0.001<br>0.39	 0.009<br>1.13
PDB Code	8C05	

Table S3.
Sample details, data collection and analysis, and modelling results from SAXS.

**Sample details**	**Dark-state *Ms*LadC**	**Light -tate *Ms*LadC**	**Dark -tate NT-*Ao*LadC**	**Light -tate NT-*Ao*LadC**
Organism	*Methylotenera sp.*		*Aquella oligotrophica*	
Expression source	*E. coli* BL21 (DE3)		*E. coli* BL21 (DE3)	
Uniprot ID	A0A2S5LZS0		A0A2I7N2Y9	
Extinction coefficient ε_{280} (cm^{-1} M^{-1})	30,370 + 19,000	30,370	20,400 + 19,000	20,400
Partial specific volume from chemical composition (cm^3 g^{-1})	0.7501		0.752	
Mw from chemical composition (Da)	36,197		33,448	
Solvent	10 mM Tris, pH 8.0, 50 mM NaCl, 2 mM $MgCl_2$		10 mM Tris, pH 8.0, 500 mM NaCl, 2 mM $MgCl_2$	
Concentration	0.8 $^{mg}/_{mL}$	0.8 $^{mg}/_{mL}$	0.9 $^{mg}/_{mL}$	0.9 $^{mg}/_{mL}$
Injection volume (µL)	50	50	50	50
**Data collection**				
Instrument	BioSAXS BM29 beamline with Pilatus3 X 2M detector			
Exposure time per frame (s)	1			
# of frames	10			
Wavelength (Å)	0.99			
Capillary temperature (°C)	20			
q measurement range (nm^{-1})	0.063 - 5.21			
Transmission	70%	100%	100%	100%
Sample configuration	Batch mode			
**Analysis software**				
Data processing	RAW (Windows version 2.1.2) & ATSAS GNOM Version 5.0 (r14254)			
Data modeling	ATSAS GASBOR 2.3p (r14885) on Debian 11			
Applied symmetry / Repetitions	P2 in gasborp / 15			
Graphic representation	ChimeraX			
**Structural parameters**				
Guinier analysis				
I(0)	61.13 ± 0.13	66.91 ± 0.18	47.77 ± 0.13	48.97 ± 0.11
R_g (nm)	2.90 ± 0.01	3.54 ± 0.02	3.37 ± 0.02	3.42 ± 0.01
qR_g limits	0.53 - 1.24	0.44 – 1.23	0.52 – 1.27	0.43 – 1.27
P(r) analysis				
I(0)	61.25 ± 0.15	67.17 ± 0.2	48.18 ± 0.17	49.55 ± 0.16
R_g (nm)	2.93 ± 0.01	3.64 ± 0.02	3.48 ± 0.02	3.61 ± 0.03
Dmax (nm)	11.1	13.8	13.6	15.7
Porod Volume (V_P) (Å^3)	147,016	176,919	165,175	166,491
Mw from V_P (kDa)	75.9	87.3	82.3	82.7
Mw from Vc (kDa)	65.7	71.3	71.7	70.8
**Modelling results**				
q range for fitting (nm^{-1})	0.181-2.763			
Final χ^2 against raw data	0.999			
SASBDB	SASDSC5	SASDSD5	SASDSA5	SASDSB5
Experiment DOI	https://doi.esrf.fr/10.15151/ESRF-ES-1057335618			

Table S4.
Primers for site-directed mutagenesis of *Ms*LadC and deletion of N-Terminus of *Ao*LadC.

	fw primer	rv primer
*Ms*LadC-Q122L	5' - AATCAGACCCTGCATGAAG AAAACCATCTGCTGAAAAGCAAC	5' - TCATGCAGGGTCTGATTCAGAATAAC CAGTGCGCTAACATCTTTCTGAATG
*Ms*LadC-N133V	5' - CTGCTGAAAAGCAACAAAGAGATGCT GGAATATCTGGTGAATATTGATGCACTG	5' - ATCTCTTTGTTGCTTTTCAGCAG ATGGACTTCTTCATGCAGGGTCTG
*Ms*LadC-K140V	5' - CATCTGCTGAAAAGCAACGTGGA GATGCTGGAATATCTGGTGAATATTGTG	5' - GTTGCTTTTCAGCAGATGGTT TTCTTCATGCAGGGTCTGATTCA
*Ms*LadC-D44S/E46K	5' - CCGTATGACCGGTTATAGCGCAAAGG AAGTGATTGGTAAAAATTGCCGTTTTC	5' - ATAACCGGTCATACGGGTAAA TGCATCATTAACAAAAACCAGCG
*Ms*LadC-R174S	5' - TGGAAACTGGCAAGC AGC CAT ATCAATACCATTACCATCTTCATGATC	5' - GCTTGCCAGTTTCCACTGAAT AACCAGCTGATCTTCCAGAAAAC
NT-*Ao*LadC	5' GGTGGCGGTGCCATGGCAATC ATCTATGGCTTTGATAGCAGC	5' GGCACCGCCACCGCCACCGC CCTGAAAATAAAGATTCTC

Table S5.
Buffer composition.

	Storage buffer	Reaction buffer
*Ao*LadC and *Dp*LadC (short linker group)	10 mM HEPES, pH 7.0, 500 mM NaCl, 2 mM $MgCl_2$	10 mM Tris, pH 8.0, 50 mM NaCl, 10 mM $MgCl_2$
*Ms*LadC and *Sf*LadC (long linker group)	10 mM Tris, pH 8.0, 50 mM NaCl, 2 mM $MgCl_2$	10 mM Tris, pH 8.0, 50 mM NaCl, 10 mM $MgCl_2$

Table S6.
Crystallization reservoirs composition.

	***Ms*nat.**	***Ms*SAD**
Additives	0.1 M Sodium formate; 0.1 M Ammonium acetate; 0.1 M Sodium citrate tribasic dihydrate; 0.1 M Potassium sodium tartrate tetrahydrate; 0.1 M Sodium oxamate	
Buffer system	0.1 M Sodium HEPES; MOPS (acid), pH 7.5	0.1 M Tris (base); BICINE, pH 8.5
Precipitant mix	20% v/v Glycerol; 10% w/v PEG 4000	20% v/v Ethylene glycol; 10% w/v PEG 8000

Table S7.

HDX data summary. Each time point was prepared in biological triplicates.

	Dark-state	Light-state	Common peptides
HDX reaction details	10 mM HEPES, 50 mM NaCl, 2 mM $MgCl_2$ pD = 7.0, 20 °C	10 mM HEPES, 50 mM NaCl, 2 mM $MgCl_2$ pD = 7.0, 20 °C	
HDX time course (s)	10, 45, 180, 900, 3600	10, 45, 180, 900, 3600	
HDX control samples	Unlabeled control (dark-state)	Unlabeled control (dark-state)	
Back-exchange (mean / IQR)	not measured		
# of Peptides	139	115	100
Sequence coverage	98%	96%	95%
Average peptide length / Redundancy	9 / 3.9	8 / 2.9	8 / 2.6
Replicates	3	3	
Repeatability (average standard deviations for all time points – 10, 45, 180, 900, 3600 s, respectively)	0.11, 0.07, 0.12, 0.12, 0.14	0.12, 0.14, 0.15, 0.12, 0.15	
Significant differences in HDX (ΔHDX > X D)	0.3 D		

Supplementary Code. Script generates folded PDB structure for *Ms*LadC protein dimer. Requirements and run description are provided as comments in the file.

Supplementary HXD Data. HDX data table includes all the peptides detected in both dark and illuminated state. It provides the peptide start and end numbering and sequence, peptide monoisotopic mass, chromatographic retention time, mean deuterium uptake and the standard deviation, for each labeling time.
